# Relational practice in health, education, criminal justice, and social care: a scoping review

**DOI:** 10.1186/s13643-023-02344-9

**Published:** 2023-10-13

**Authors:** Gary Lamph, Rebecca Nowland, Paul Boland, Jayn Pearson, Catriona Connell, Vanessa Jones, Ellie Wildbore, Danielle L Christian, Catherine Harris, Joanne Ramsden, Kathryn Gardner, Nicola Graham-Kevan, Mick McKeown

**Affiliations:** 1 School of Nursing and Midwifery, Edge Hill University, Lancashire Ormskirk, UK; 2 School of Nursing and Midwifery, University of Central Lancashire, Preston, UK; 3 IMPlementation and Capacity Building Team (IMPaCT), Applied Health Research Hub (AHRh), University of Central Lancashire, Preston, UK; 4 Criminal Justice Partnership, University of Central Lancashire, Preston, UK; 5 Salvation Army Centre for Addiction Services and Research, Faculty of Social Sciences, University of Stirling, Stirling, UK; 6 School of Psychology and Counselling, The Open University, Milton Keynes, UK; 7 Sheffield Health and Social Care NHS FT, Sheffield, UK; 8 Health Technology Assessment Unit, University of Central Lancashire, Preston, UK; 9 Leeds and York Partnership Foundation Trust, Leeds, UK; 10 School of Psychology and Humanities, University of Central Lancashire, Preston, UK

**Keywords:** Relational practice, Enabling environments, Relational approach, Health, Social care, Justice, Education

## Abstract

**Background:**

Establishing and maintaining relationships and ways of connecting and being with others is an important component of health and wellbeing. Harnessing the relational within caring, supportive, educational, or carceral settings as a systems response has been referred to as relational practice. Practitioners, people with lived experience, academics and policy makers, do not yet share a well-defined common understanding of relational practice. Consequently, there is potential for interdisciplinary and interagency miscommunication, as well as the risk of policy and practice being increasingly disconnected. Comprehensive reviews are needed to support the development of a coherent shared understanding of relational practice.

**Method:**

This study uses a scoping review design providing a scope and synthesis of extant literature relating to relational practice focussing on organisational and systemic practice. The review aimed to map how relational practice is used, defined and understood across health, criminal justice, education and social work, noting any impacts and benefits reported. Searches were conducted on 8 bibliographic databases on 27 October 2021. English language articles were included that involve/discuss practice and/or intervention/s that prioritise interpersonal relationships in service provision, in both external (organisational contexts) and internal (how this is received by workers and service users) aspects.

**Results:**

A total of 8010 relevant articles were identified, of which 158 met the eligibility criteria and were included in the synthesis. Most were opinion-based or theoretical argument papers (*n* = 61, 38.60%), with 6 (3.80%) critical or narrative reviews. A further 27 (17.09%) were categorised as case studies, focussing on explaining relational practice being used in an organisation or a specific intervention and its components, rather than conducting an evaluation or examination of the effectiveness of the service, with only 11 including any empirical data. Of the included empirical studies, 45 were qualitative, 6 were quantitative, and 9 mixed methods studies. There were differences in the use of terminology and definitions of relational practice within and across sectors.

**Conclusion:**

Although there may be implicit knowledge of what relational practice is the research field lacks coherent and comprehensive models. Despite definitional ambiguities, a number of benefits are attributed to relational practices.

**Systematic review registration:**

PROSPERO CRD42021295958

**Supplementary Information:**

The online version contains supplementary material available at 10.1186/s13643-023-02344-9.

## Background

While there is not a clearly outlined definition of relational practice it is generally understood as an approach that gives priority to interpersonal relationships in both in relation to external (organisational contexts) and internal (how this is received by workers and service users) aspects. It is the foundation upon which effective interventions are made, and it forms the conditions for a healthy relational environment [1]. Notions of relational practice are not new. Systematised relational, psychosocial approaches to mental health care have a lengthy heritage, for example in the development of therapeutic communities [2]. Building on such traditions, Haigh and Benefield [3] describe the importance of relational practice in working toward a unified model of human development, with cross-sector implications. Within their work, they describe the importance of a ‘whole-person, whole-life perspective in the field of human relations’ with the quality of relational activity defined as being central to positive human outcomes and effective service provision [3]. The term relational practice is increasingly described and applied across different service contexts, including health, education, criminal justice and social work. The ways in which relational practice is described varywidely but include Psychologically Informed Environments, Enabling Environments and Psychosocial Environments, as well as other environments and work practices that use the relational practice label to describe their provision.

The importance of relationships and ways of connecting and being with others cannot be underestimated with respect to positive health, wellbeing and other outcomes (such as mental health recovery, overcoming social challenges, rehabilitation in criminal justice services and learning). However, practitioners, people with lived experience, academics and policy makers have not yet articulated a shared understanding of relational practice [3]. Consequently, there is a potential for interdisciplinary and interagency miscommunication, as well as a risk of policy and practice being disconnected. Further, inconsistency in terminology between disciplines is likely to result in separate knowledge bases being developed in parallel, complicating and compromising transfer of evidence into practice across fields.

While there are some systematic or scoping reviews of relational practice in specific service provision, such as acute care settings [4] or after-school provision [5], to date, there has been no comprehensive synthesis or mapping of the extant relational practice literature, across disciplines. Largely absent from the literature are reviews that focus on organisational practice using relational approaches, rather than focused on individual and/or therapeutic relationships. This scoping review maps and combines literature across a range of disciplines (Health, Education, Criminal Justice, and Social Care/Work) to provide clarity and direction, charting and summarising existing understandings. As we aimed to examine evidence from disparate or heterogeneous sources, rather than seeking only the best evidence to answer a specific question, a scoping review methodology was considered appropriate [6]. This methodology enables an examination and synthesis of the extent, range and nature of research on relational practice across health, criminal justice, social care/work and education, to inform future systematic reviews, and to identify gaps in the literature [7]. The four chosen sectors (health, criminal justice, social care/work and education) were included as these are all people facing public service contexts where relationships and relational practices are of crucial importance. We used the service provision being provided or discussed in the papers as criteria for inclusion rather than academic discipline.

The review focuses specifically on the relational practice used in an organisational context rather than in one-to-one approaches (i.e., individualised therapeutic approaches). We used the following definition: relational practice from a systemic and organisational perspective, defined as practice and/or intervention that prioritises interpersonal relationships in service provision, in both external (organisational contexts) and internal (how this is received by workers and service users) aspects. This approach was adopted owing to the ambitious and broad scope of this review, but also to add a focus as there is a wide range of relational approaches that are focused upon individualised interventions, such as therapeutic relationships and other evidence-based therapeutic/psychological approaches, but less is known about relational approaches at an organisational level. Within the review, we also scoped the extant literature for any reported impacts and benefits of relational practice.

### Research question

How is relational practice used, defined and understood across different academic disciplines, professional practices and contexts, focussing on Health, Education, Criminal Justice, and Social Care/Work, and what are its reported impacts and benefits?

## Method

This scoping review was conducted in accordance with the best practice guidance and reporting items for the development of scoping reviews [6]. The Preferred Reporting Items for Systematic reviews and Meta-analyses extension for Scoping Reviews (PRISMA-ScR) is provided in Additional file 1. Prior to commencement, the review protocol was registered with PROSPERO (registration number: PROSPERO2021CRD42021295958) and is available at: https://www.crd.york.ac.uk/prospero/display_record.php?ID=CRD42021295958.

A multi-disciplinary core research team was brought together made up of academics, clinicians and people with lived experience of service provision with representation from health, education, social care/work and criminal justice experiences. A steering group committee was also convened with similar representative experience. This group provided oversight of the project, informed by subject matter expertise complimenting cross-sector and occupational/lived experience among the membership and supported with the search strategy, identifying search terms and the synthesis of the literature.

### Search strategy

Searches were conducted on eight electronic databases (EPIC, SocIndex, Criminal Justice Abstracts, Education Abstracts, PsycInfo, CINAHL, Ovid MEDLINE and Criminal Justice Database) on 27 October 2021. Keywords for database searches included the following:

- Relational focussed OR relational based OR relational work OR Relational social work OR Relational centred OR Relational centered OR relational practice* or relational informed OR relational theory OR relational approach* OR relational perspective* OR relational model OR relational strategy or relational strategies OR relational environment* or relational justice* OR relational education* or relational health OR relational therapy OR relational thinking OR relational inquiry OR relationship focussed OR relationship based practice OR relationship informed OR interpersonal system OR interpersonal environment* OR interpersonal practice OR interpersonal approach* or interpersonal perspective OR interpersonal strategy or interpersonal strategies OR psychosocial environments OR enabling environments

The full search strategy for each database is included in Additional file 2.

### Inclusion and exclusion criteria

The inclusion and exclusion criteria for the review are displayed in Table 1. In order to encompass a broad range of approaches, the relational practice was broadly defined as practices and/or interventions that prioritise interpersonal relationships in service provision, in relation to both external (organisational contexts) and internal (how this is received by workers and service users) aspects. Articles were included where relational practice was seen as the foundation upon which effective interventions are made and form the conditions for a healthy relational environment. Because the focus of this review was on organisational processes, articles exclusively about the therapeutic relationship and/or therapeutic approaches were only included if they informed a systemic and organisational approach.

**Table 1:** Inclusion and exclusion criteria

	Inclusion	Exclusion
Population	Any patients or service users without age restriction (e.g., including children and youth) accessing face-to-face health, education, justice or social care/social work	Computer-facing services—e.g., virtual platform interventions (Telecare, etc.), artificial intelligence, relational data bases. Human–computer interaction
Concept	Relational practice from a systemic and organisational perspective, defined as practice and/or intervention that prioritises interpersonal relationships in service provision, in relation both to external (organisational contexts) and internal (how this is received by workers and service users) aspects.	Studies/articles purely about the therapeutic relationship/alliance Studies of interventions/work practices/ Service Provision where the focus is not specifically on the relational component Studies/articles about an evidence-based or therapeutic interaction linked to a psychological intervention or focussed solely on group/one-to-one interactions
Context	People facing services across and the following sectors: Education *(including any type of education provision, i.e. school, college, university)*, Health (*any health service)*, Criminal Justice (*e.g. Liaison and Diversion, Prisons, Probation, Offender Personality Disorder, YOT, Police, Special Hospital),* and Social Care/Work (including third sector organisation provision)	Any services/organisations outside of the 4 defined sectors. For example, studies examining exclusively business and work focussed.

All types of studies (qualitative, quantitative and mixed methods), conceptual or theoretical papers/reports and all types of reviews (i.e., systematic, scoping, meta-analysis) were included, reported in peer-reviewed journals, grey literature and book and book chapters fully available online. Only articles published in English were included; however, there was no limit on the country of origin. Only articles published from 2000 were included to focus on current/recent practice and service delivery.

### Study/paper selection

All records identified from the database searches were downloaded to EndNote and duplicates were removed using Systematic Review Accelerator [8]. Any remaining citations were transferred to Rayyan for screening, and any further duplicates identified were removed. Title and abstract screening were conducted in Rayyan independently by five reviewers (PB, RN, MM, GL, JP), with 20% of the papers screened independently by at least two reviewers. Inter-rater reliability was 84.09% at the title and abstract stage. Once title and abstract screening were complete, selected full-text papers were sourced and checked against inclusion criteria by six reviewers independently (PB, RN, MM, GL, JP), with at least 20% of the papers screened by at least two reviewers. An inter-rater reliability of 85% was achieved at the full-text screening stage. Reasons for exclusion were noted at this stage. Agreement at all stages was made by consensus, and any disagreements regarding inclusion were discussed with a third member of the research team where necessary.

### Data extraction

Data were extracted from all selected texts using a data extraction sheet designed by the research team in collaboration with the steering group committee. The data extraction tool was piloted and refinements made. Following this authors completed data extraction for a sample of 10 studies as sufficient agreement was reached the authors then applied to tool to the remaining studies independently. Data extraction included key study/article characteristics (e.g., country), people facing service type, sector type, aims of study, study/article type, including key information for empirical studies (i.e., participants, design, data collection methods), underlying theories, key terms and definitions of relational practice and reported impacts and benefits. The research team collectively carried out calibration testing of the tool with a sample of articles prior to the assignment of independent data extraction of research team members [9].

### Data synthesis

Data extracted from selected articles was charted, and a mapping of the scope of the literature was conducted using narrative synthesis. Narrative synthesis is an often-used approach within systematic and scoping study literature reviews. This approach enables the synthesis of large bodies of literature and looks to explore the relationships in the dataset collected and analyse commonalities, conflicts and relationships that assist us to reach conclusions and make recommendations for practice [10]. Consultation with the steering group committee throughout the data synthesis stage supported the interpretation and synthesis of the review findings.

## Results

**Fig. 1:**
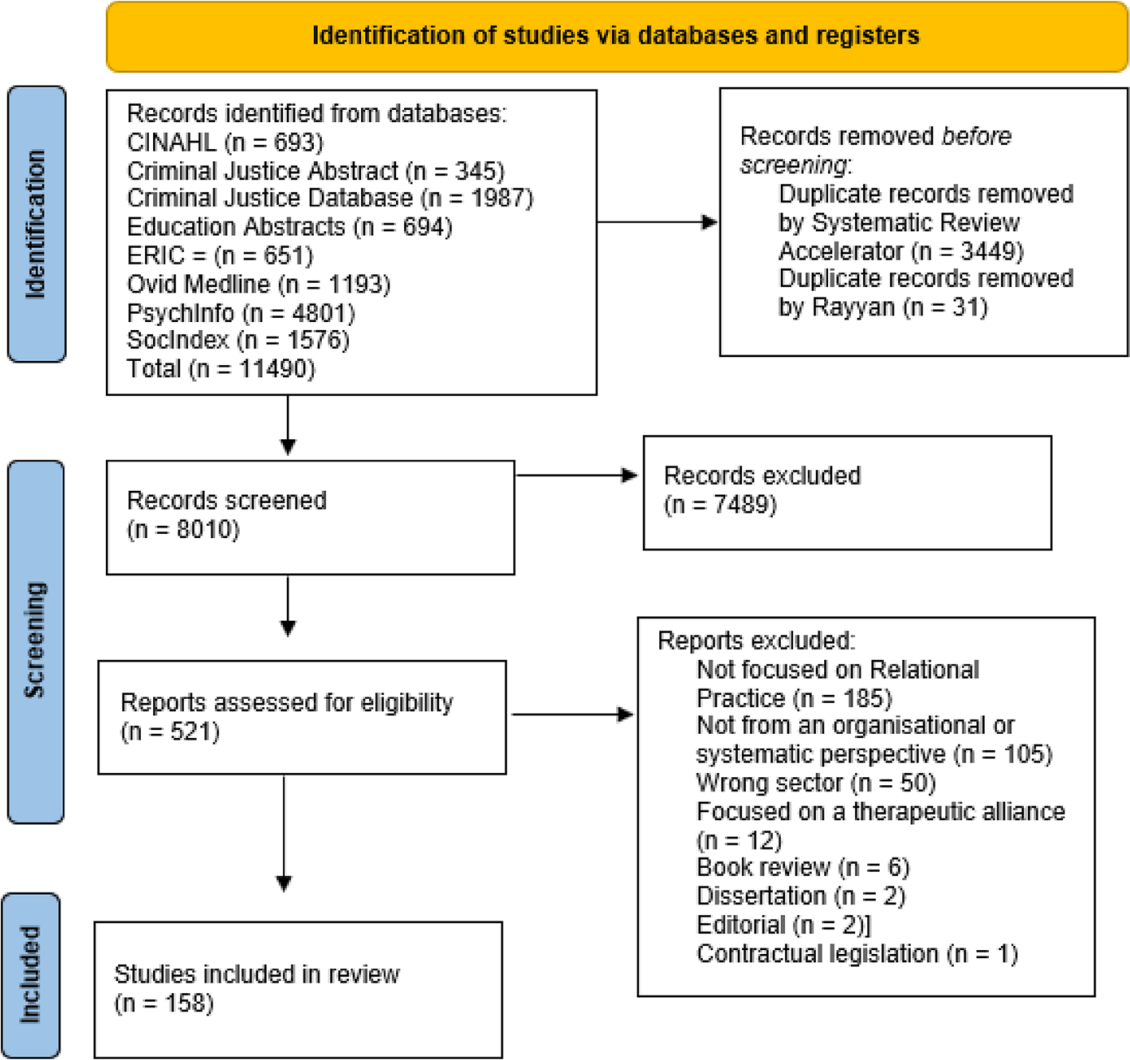
PRISMA flowchart

The results of the systematic search and screening process are displayed in a PRISMA flow chart (see Fig. 1). A total of 11,490 articles were initially identified from database searches. After the removal of duplicates, 8010 were retained, and 521 remained for full-text review. Overall, 158 articles met the eligibility criteria and were included in the synthesis. Table 2 displays the characteristics of the studies/articles included.

**Table 2:** Characteristics of included articles/studies

First author	Year	Country^a^	Service users	Professionals	Sector type	Specific service type	Aim of study/paper	Study type
Aggett [11]	2019	UK	Child and Adolescents Mental Health (CAMHS) service users	Clinical psychologists	Health	CAMHS	Propose a model of risk management that moves away from an overemphasis on ‘technical’ approaches to ensuring that this is balanced by organisations supporting ‘relational’ approaches and further, ‘relational-collaborative’ approaches	Opinion piece/theoretical argument
Anderson [12]	2016	US	Care home residents	Care aides	Health	Nursing homes	Exploring the complexities of care; working environments; and knowledge, skills, and efforts of care aides who work in nursing homes.	Qualitative
Andrews [13]	2018	Canada	Mothers with substance abuse	Clinicians/social workers/academics	Health	Multi-sector including health, social care	Explore mothers’ service use at breaking the cycle, an early intervention and prevention program for pregnant and parenting women and their young children in Toronto, Canada.	Quantitative
Andrews [14]	2019	Canada	Community-based projects	Academic researchers	Health	Community projects supporting vulnerable families	Describes two approaches integrated into a multiyear, multiphase research and evaluation initiative supporting the health and well-being of vulnerable families: (1) a relational approach and (2) a trauma-informed approach; specific strategies and key considerations used are outlined.	Opinion Piece/theoretical argument
Appleby [15]	2020	New Zealand	Young offenders	Social workers	Criminal justice	Youth offending and mental health provision	Focuses on the social work contribution to service improvement by reflecting on the establishment of the first youth forensic forum in Aotearoa New Zealand, to improve mental health assessment experiences for young people within youth justice residences.	Opinion piece/theoretical argument
Arnkil [16]	2015	Finland	Psychotherapy clients and students	Psychotherapy/family therapists	Health	Mental health	An analysis of the use of open dialogicity in psychotherapy and juxtaposes it with education in order to find common dialogical elements in all relational practices	Opinion piece/theoretical argument
Asakura [17]	2018	Canada	Student social workers	Experienced social workers	Social care/work	Field work coordination for trainee social workers	Conceptualizes field coordination as a negotiated pedagogy in which the coordinators navigate complex and often competing needs among students, field agencies, and social work practice.	Opinion piece/theoretical argument
Bainbridge [18]	2017	UK	Female offenders	Forensic clinicians	Criminal justice	Women in custody	Considers the development of the therapeutic environment of a PIPE (psychologically informed planned environments) Unit and in particular its translation for women in custody	Qualitative
Barrett-lennard [19]	2011	Australia	Psychotherapy clients	Psychotherapists	Health	Mental health	Stresses the connectedness of human lives, and views our life process and consciousness as relational in its essence	Opinion piece/theoretical argument
Barrow [20]	2021	UK	Young people who have experienced child sexual exploitation (CSE)	Clinical psychologists	Health	Mental health, young people, CSA/CSE	Service evaluation: explored viewpoints of key stakeholders, such as young people and frontline staff, about CSE services	Quantitative
Bennett [21]	2017	UK	Offenders	Prison governor, clinical service head	Criminal justice	Prison-based democratic therapeutic communities	Describe the work of HMP Grendon, the only prison in the UK to operate entirely as a series of democratic therapeutic communities and to summarise the research of its effectiveness.	Case study/report
Bennett [22]	2018	UK	Offenders	Prison governor, clinical service head	Criminal justice	Prison-based democratic therapeutic communities	Consider how the more positive social climates found in democratic therapeutic communities are constructed and how these practices can be replicated in other settings	Opinion piece/theoretical argument
Berzoff [23]	2006	US	Social work students	Lecturers	Education	Masters course in end-of-life care for social work students	Describes the first post-master’s program in the US in end-of-life care for social workers	Case study/report
Bjornsdottir [24]	2018	Iceland	Older persons receiving care at home	Senior nurses/academics	Health	Home care nursing for elderly people	Enhance knowledge and understanding of the nature of home care nursing practice.	Qualitative
Blagg [25]	2018	Australia	Young people with fetal alcohol spectrum disorder (FASD)	Criminology academics	Criminal justice	Youths with FASD in the justice system	Reports on a study undertaken in three Indigenous communities in the West Kimberley region of Western Australia (WA) intended to develop diversionary strategies for young people with fetal alcohol spectrum disorder (FASD).	Mixed methods
Blumhardt [26]	2017	New Zealand	Children and family	Academics; anti-poverty non-governmental organisation	Social care/work	Vulnerable, excluded families in poverty	Posits the radical practice of anti-poverty organisation ATD Fourth World in England (where child protection is characteristically risk-averse, individualised and coercive), as an alternative for work with families experiencing poverty and social exclusion	Qualitative
Bøe [27]	2019	New Zealand	Children in child protection institutions	Milieu therapists	Social care/work	Child protection institutions	Examine factors described by milieu therapists as significant for relational work with youth placed in institutions	Qualitative
Boober [28]	2005	US	Incarcerated women (ready for parole)	Staff members involved in the transition programme	Criminal justice	Prison context, re-entry to society	Describe the Maine Re-entry Network transition program at the Women's Center in Windham, Maine,	Case study/report
Booth [29]	2012	US	Adult learners	University lecturers	Education	University	Discuss the characteristics of working with adult learners relating to interpersonal boundaries	Opinion piece/theoretical argument
Boschki [30]	2005	Germany	Student	Teacher	Education	Religious education (school context)	Discusses the possibilities and chances of a relational approach to religious education	Critical/narrative review
Bridges [31]	2014	UK	Older people	Nurses	Health	Elderly residential care	Propose the use of a novel implementation programme designed to improve and support the delivery of compassionate care by health and social care teams.	Opinion piece/theoretical argument
Bridges [32]	2017	UK	Acute care patients	Nursing staff, managers	Health	Nursing acute care	Identify and explain the extent to which Creating Learning Environments for Compassionate Care (CLECC) was implemented into existing work practices by nursing staff, and to inform conclusions about how such interventions can be optimised to support compassionate care in acute settings	Qualitative
Bridges [33]	2020	Various	Elderly inpatients	Nursing staff	Health	Elderly inpatient hospital care	To synthesise qualitative research findings into older people’s experiences of acute healthcare	Systematic review
Brown [34]	2018	Ireland	Children and young people in care	Residential care home staff	Social care/work	Residential childcare	Explores the views and experiences of residential care workers regarding relationship‐based practice.	Qualitative
Bunar [35]	2011	Sweden	Parents	Teachers, principals	Education	Multicultural urban schools	To outline an argument that a relational approach is needed in multicultural schools	Opinion piece/theoretical argument
Burchard [36]	2005	UK	Families	Community nurses	Health	Family nursing	A comparison of ethical principles relating to research, family nursing practice, and Foucault’s meta-ethical framework is offered	Critical/narrative review
Byrne [37]	2016	UK	Young offenders	Social workers, youth justice workers	Criminal justice	Youth criminal justice	Consider and explore the principles that should inform a positive and progressive approach to conceptualising and delivering youth justice.	Opinion piece/theoretical argument
Cahill [38]	2016	Ireland	Young people in care	Residential care staff	Social care/work	Residential childcare	Exploring relationship-based approaches in residential childcare practice, from the perspectives of both residential childcare workers and young care leavers	Qualitative
Campbell [39]	2012	India, South Africa	Community out-patients	Nurses	Health	Home-based nursing (AIDS)	Explore transformation communication by presenting a secondary analysis of two contrasting case studies using peer education with highly marginalised women in HIV/AIDS management.	Case study/report
Carpenter [40]	2015	US	Students, faculty members	Higher education leaders	Education	University	Examine the strategic organization-public dialogic communication practices of universities in the USA	Qualitative
Celik [41]	2021	Germany	Students	Teachers	Education	Secondary school	Explain a relational framework that ties the concepts of institutional habitus, field and capital, and investigate how a secondary school improves the educational engagement of working-class, second-generation Turkish immigrant youth in Germany.	Case study/report
Cheung [42]	2017	Hong Kong	Clients	Social workers	Social care/work	Social work	Drawing from the experiences of community development projects in rural Hong Kong, discuss how guanxi among social workers, clients and other stakeholders in Chinese communities might challenge the professionalism of social work and breach the boundaries of social work relationships.	Opinion piece/theoretical argument
Cleary [43]	2012	Various	Mental health inpatients	Mental health nurses	Health	Acute mental health inpatient care	Identify, analyse and synthesize research in adult acute inpatient mental health units, which focused on nurse-patient interaction.	Systematic review
Cleland [44]	2021	UK	Parent involvement with students	Teachers	Education	Compulsory education only	Explores examples of parent-school relations which impact positively on parents, regarding empowerment, parent voice and social capital.	Systematic review (meta-ethnography)
Coleman [45]	1999	Greece	Families	Training teachers	Education	Early years education	Justifying a family involvement training course	Opinion piece/theoretical argument
Collier [46]	2010	UK	Older people with mental health difficulties	Mental health professionals	Health	Older Persons Mental Health	Exploration of ethics in the context of older persons mental health care	Opinion piece/theoretical argument
Collinson [47]	2019	UK	Substance misuse	Substance misuse workers	Health	Recovery and Substance Misuse	Shares an asset-based community model highlighting the strong dynamic relationship between the key components of recovery capital and represents a foundation for community and therapeutic-level interventions for building recovery capital.	Opinion piece/theoretical argument
Conradson [48]	2003	UK	Community centre service users	Community centre workers	Social care/work	Community drop-in centres—Brexton House Bristol	Explores the ways in which drop-in centres may at times function as spaces of care in the city, focussing upon social relations within the drop-in space and the various subjectivities that emerge in this relational environment.	Qualitative
Cranley [49]	2020	Canada	Older residents	Nursing staff	Health	Older persons residential Care	Explore shared decision-making among residents, families and staff to identify relevant strategies to support shared decision-making in LTC.	Qualitative
Creaney [50]	2014	UK	Youth services	Youth workers	Criminal justice	Youth Justice	Critical Review of the “position of relationship-based practice” in youth justice, in particular looking at how “effective programmes” seem to have been given heightened importance over “effective relationships”	Critical/narrative review
Creaney [51]	2015	UK	Youth services	Youth workers	Criminal justice	Hard to engage young people	Examination of how youth justice practice could become more participatory and engaging, particularly with those who are "involuntary clients" or in other words difficult to engage.	Opinion piece/theoretical argument
Creaney [52]	2020	UK	Youth services	Youth workers	Criminal justice	Youth Justice	Explore young people’s experiences of youth justice supervision with particular reference to the efficacy of participatory practices	Qualitative
Cuyvers [53]	2013	Belgium	Social work undergraduates	Social work lecturers	Education	Social work education	Describe a relational practice approach embedded in appreciative inquiry in social work education	Opinion piece/theoretical argument
Daly [54]	2020	UK, New Zealand	Students	Teachers	Education	Schools and Systems	Examines schools as ‘systems’ in which teachers learn; conceptualising schools from an ecological perspective, the relations among all stakeholders are brought into focus.	Opinion piece/theoretical argument
Daniel [55]	2018	Canada	Children and young people	Youth workers	Social care/work	Children and youth services	Expand upon Garfat’s [56] exposition and ask that we rethink our understandings and practice in the field of CYC when we incorporate sites of diversity.	Opinion piece/theoretical argument
Davies [57]	2019	UK	Offenders	Probation and prison	Criminal justice	Probation and Prison	Examines the progress in the introduction of the Enabling Environments (EE) standards across seven sites (four Approved Premises and three prisons)	Case study/report
Deery [58]	2008	UK	Prenatal and postnatal women/birthing people	Midwifery	Health	Community-based Midwifery	Examines community midwives’ experience of linear time during the third phase of a 3-year action research study, seeking to compare and contrast the ways in which they experienced this temporal framework, individually and organizationally, in their clinical practice.	Qualitative
Defrino [59]	2009	US	Patients	Nurses	Health	Nursing not specific	Discusses the theory of the relational work of nurses derived from a psychodynamic theory of the relational practices of women and the workplace.	Opinion piece/theoretical argument
Dewar [60]	2013	UK	Older people, staff and relatives	Medical staff not defined	Health	Acute hospital setting	Actively involve older people, staff and relatives in agreeing a definition of compassionate relationship-centred care and identify strategies to promote such care in acute hospital settings for older people.	Qualitative
Doane [61]	2002	Canada	Student nurses	Nursing lecturers	Education	Nursing education	Discusses the pedagogical value of interpretive inquiry for the teaching–learning of relational practice.	Opinion piece/theoretical argument
Doane [62]	2007	Canada	Patients	Nurses	Health	General nursing	Critically examine the concept of obligation in nursing practice, and using a relational understanding, suggest 3 obligations underlying nursing relationships, proposing that responsive, compassionate, therapeutic relationships, and ethical and competent nursing practice are integrally connected, and that relational inquiry can support the enactment of both.	Opinion piece/theoretical argument
Dupuis [63]	2012	US	People living with dementia	Staff providing dementia care	Health	Dementia care	Description of new relational approach, that views persons with dementia as equal partners in dementia care, support and formal services: ‘authentic partnerships.	Opinion piece/theoretical argument
Durocher [64]	2019	Canada	Older adults	Healthcare professionals	Health	Older adult inpatient rehabilitation unit	To discern relational approaches adopted by families in planning for the discharge of older adults from inpatient settings and how they inform practice in discharge planning with older adults.	Case study/report
Ellery [65]	2010	New Zealand	Secondary school children years 7–11	Teachers	Education	Secondary school	Discover how RTLB (Resource Teachers: Learning and Behaviour) can effectively support secondary teachers to enhance inclusive classroom practices.	Qualitative
Elliott [66]	2011	Australia	Members of public (victims of crime)	Police	Criminal justice	Policing	Test a relational model of authority in victim-police interactions and examine what perceived antecedents of procedural justice in contacts with the police mean for victims of crime.	Mixed methods
Elwyn [67]	2021	US	Patients	Healthcare professionals	Health	Healthcare in general	Present an argument that the process commonly described as shared decision making involves work that is cognitive, emotional, and relational, and particularly if people are ill, should have the underpinning goal of restoring autonomy.	Opinion piece/theoretical argument
Emmamally [68]	2018	South Africa	Patients and their families	Emergency dept professionals	Health	Emergency care	To describe the adherence of emergency healthcare professionals to family-centred practices in some emergency departments	Quantitative
Emmamally [69]	2020	South Africa	Patients and their families	Emergency dept professionals	Health	Emergency care	Describe Health Care Providers’ perceptions of relational practice with families in emergency department contexts	Qualitative
Emmamally [70]	2020	South Africa	Families	Emergency dept professionals	Health	Emergency care	To describe families’ perceptions of relational practice when interacting with health care professionals in emergency departments in the South African context.	Qualitative
Ferguson [71]	2020	UK	Service users	Social work teams, family support workers	Social care/work	Child protection	Examine what social workers actually do, especially in long-term relationships.	Mixed methods
Finkelstein [72]	2005	US	Women with alcohol/drug use and mental health disorders with histories of violence	Women Embracing Life and Living (WELL) Project providers	Health	Substance use/violence prevention	Describe the organisation and delivery of a service based on the relational model of women’s development	Case study/report
Fitzmaurice [73]	2015	US	Academic staff/university	Academic staff/university	Education	University	Describe a program of faculty support that places trust and community-building at the center of its efforts.	Case study/report
Fortuin [74]	2007	US	Women offenders	Corrections and voluntary agency staff	Criminal justice	Rehabilitation of offenders	Describes transition, reunification and re-entry programme for female offenders in Maine	Case study/report
Frelin [75]	2014	Sweden	Secondary school students	Teachers and other staff	Education	Secondary school	Introduces a theoretical framework for studying school improvement processes using concepts from spatial theory, in which distinctions between mental, social and physical space are applied makes for a multidimensional analysis of processes of change.	Opinion piece/theoretical argument
Frost [76]	2008	UK	Social care service users	Social workers	Social care/work	Social work services, social work education, research	Examining how the current re-emergence of psychosocial theory, mainly emanating from sociology, is useful for informing social work theory.	Opinion piece/theoretical argument
Gharabaghi [77]	2008	Canada	Young people and families	Child and youth care practitioners	Social care/work	Child and youth care	Explores the professional issues of relationships within child and youth care practice.	Opinion piece/theoretical argument
Gharabaghi [78]	2008	Canada	Young people and families	Youth workers	Social care/work	Youth work	Highlight five dialectical processes within relational youth work in the hopes that we might collectively engage not only in the celebration of our concept, but also in a serious contemplation of its pitfalls.	Opinion piece/theoretical argument
Gill [79]	2020	UK, US	Students	Teachers	Education	Schools	Describe a relational perspective for educational evaluation	Opinion piece/theoretical argument
Giller [80]	2006	US	Various service provider practitioners	Educators	Education	Practitioner education curriculum development	Discuss how, Risking Connection teaches the philosophy of relational therapy and how collaborative relationships have nurtured the development, application, and follow-up of Risking Connection.	Opinion piece/theoretical argument
Goddard [81]	2021	US	Nursing students	Nurse educators	Education	Nurse education	A call to action for trauma awareness in nursing education, aiming to guide nursing educators, researchers, and leaders in support, retention, and building foundational skill sets in a now traumatized nursing student population.	Opinion piece/theoretical argument
Grimshaw [82]	2016	UK	Older adults	Health and social care workers	Health	Older peoples care	Scoping literature review of the H&SC and broader management literature to identify and extract important behaviours, processes and practices underlying the support of high-quality relationships.	Scoping review
Haigh [3]	2019	UK	Service users	public sector workers	All sectors	General health, justice, social care and education services	To agree a better map of human development by using an iterative process of consultation with professionals and specialists in relevant disciplines, and service users	Opinion piece/theoretical argument
Haigh [2]	2013	UK	Mental health service users	Mental health professionals	Health	Mental health care	Describe the necessary primary emotional development experiences for healthy personality formation.	Opinion piece/theoretical argument
Hibbin [83]	2019	UK	School children	Teacher	Education	Primary and secondary schools	Consider how provision for children with SEND (Special Educational Needs and Disabilities) is conceptualised, operationalised and enacted	Qualitative
Holt [84]	2018	UK	Children and their families	Social workers	Social care/work	Social work with children and families	Outline how reforms to the family justice system limit the potential for social workers to engage in relationship-based work with children and their families.	Opinion piece/theoretical argument
Horowitz [85]	2015	US	Psychiatric inpatients	Mental health nurses	Health	Mental health hospitals	Argues for trauma-informed, person-centred, recovery-focused (TPR) care in psychiatric hospitals	Opinion piece/theoretical argument
Howitt [86]	2020	Australia	Community members on a public housing estate	Salvation army workers and volunteers	Social care/work	Community development	Explore the transformative potential of relational, rather than transactional, community development practices	Qualitative
Ingólfsdóttir [87]	2021	Iceland	Young disabled children and their families	Professionals providing specialised services	Social care/work	Services for disabled children and their families	Views and experiences of professionals providing specialised services to disabled children and their families.	Qualitative
Jennings [88]	2018	US	Patients	Clinical staff	Health	Bioethics	To advance the discussion of relational approaches within bioethics by an interpretive analysis of the concept of solidarity and the concept of care when seen as modes of moral and political practice	Opinion piece/theoretical argument
Jindra [89]	2020	US	Residents/community members	Community workers	Social care/work	Anti-poverty non-profit organisations	Critique of the precariousness and promise of relational work in anti-poverty organisations	Opinion piece/theoretical argument
Kanner [90]	2005	US	Adolescents with emotional, behavioural and educational issues	Teaching and support staff	Education	Educational setting for adolescents with emotional, behavioural and educational issues	Description of the relational approach of the Kanner Academy drawing to the relational ethic that derives from Gestalt field theory.	Case study/report
Kerstetter [91]	2016	US	School children	Teaching and support staff	Education	Public charter schools—'no excuses' schools	Examines the extent to which authoritarian discipline systems are necessary for success at “no excuses” schools, drawing upon qualitative research at a strategic site	Qualitative
Kippist [92]	2020	Australia	Renal care patients	Carers and staff	Health	Regional dialysis centre	Presents findings from the first of a two-part study exploring user experiences of brilliant renal care within the Regional Dialysis Centre in Blacktown (RDC-B)	Qualitative
Kirk [93]	2017	UK	Children	Social workers and team managers	Social care/work	Child Protection	Describes and evaluates an approach to social work practice, which divides levels of risk within the child in need category enabling adequate, coordinated support and oversight to be provided	Qualitative
Kitchen [94]	2009	Canada	children	Teachers	Education	Elementary school	Examines how a respectful and relational approach to teacher development can result in deep and sustained professional growth and renewal.	Case study/report
Kong [95]	2020	Hong Kong	Women who have left abusive partners	Social work practitioner-researcher	Social care/work	Domestic violence service (crisis intervention)	Provide insights for improving the local domestic violence service, whose main focus is on crisis intervention.	Qualitative
Kranke [96]	2019	US	Veterans	Military social workers	Social care/work	Veteran reintegration support	Propose a practice paradigm shift among veterans that would also focus on the attributes of “sameness” rather than differentness alone.	Opinion piece/theoretical argument
Kuperminc [97]	2019	US	Boys and Girls Clubs of America members	Programme staff	Education	After school settings	Examined associations among programmatic structures, workplace and workforce characteristics, and relational practices of program staff as they relate to young people’s ratings of their experience attending local clubs.	Quantitative
Kutnick [98]	2014	Hong Kong	School children	Teachers	Education	Primary school	Effectiveness of a relation-based group work approach adapted/co-developed by HK primary school mathematics teachers	Quantitative
Larkin [99]	2020	UK	Unaccompanied young females (UYFS)	Social workers	Social care/work	Care for female under 18 unaccompanied asylum seekers	Through a study of how UYFS and practitioners in England experienced and constructed each other during their everyday practice encounters, the potential of the practice space for creating mutual understandings and enabling positive changes is discussed	Qualitative
Laschinger [100]	2014	Canada	Patients	Nurses	Health	Hospital & community	Test a model linking a positive leadership approach and work-place empowerment to workplace incivility, burnout, and subsequently job satisfaction	Quantitative
Lees [101]	2016	UK	Workers in therapeutic communities or similar services	Senior TC clinicians, group psychotherapists	Health	Training for workers in TCS, enabling environments and similar	Describe transient therapeutic communities (TCS) and their value for training. This is a descriptive account which includes the findings of two field study evaluations, and direct participant feedback.	Case study/report
Lefevre [102]	2019	UK	Children at risk/being sexually exploited	Various (police, social work)	Social care/work	Child protection	Analysis of data from a 2-year evaluation of the piloting of a child-centred framework for addressing child sexual exploitation (CSE) in England to illuminate the dilemma between control and participation, and strategies used to address it	Qualitative
Leonardsen [103]	2007	Norway	Social work service users	Social workers	Social care/work	General social work	Differentiate individual vs. relational approaches to empowerment and argue for changes to social work standards/education	Opinion piece/theoretical argument
Lindqvist [104]	2014	Sweden	Students	Teachers	Education	Primary/secondary school	Examine the strategies used by teachers whose practice was considered inclusive	Qualitative
Ljungblad [105]	2021	Sweden	Children	Teachers	Education	Schools	Explaining Pedagogical Relational Teachership	Opinion piece/theoretical argument
Llewellyn [106]	2012	Canada	Citizens of war-affected countries	People in peace keeping institutions	Criminal justice	International peace building	Setting out a theory of relational justice	Opinion piece/theoretical argument
Lloyd [107]	2015	UK	Schools and families	Teachers	Health	Primary school obesity intervention	Overview of the conceptualisation and development of a novel obesity prevention intervention, the Healthy Lifestyles Programme	Case study/report
Macritchie [108]	2019	UK	Care experienced children	Volunteer mentors	Education	Primary and secondary school	Describe MCR pathways—mentoring programme for care-experienced young people	Case study/report
Markoff [109]	2005	US	Women with SU/MH disorder and trauma histories	Senior health providers	Health	Substance abuse/mental health	Describe the principles and strategies used to document and evaluate WELL Project implementation, and evidence of resulting systems change to support the delivery of integrated and trauma-informed services for women with co-occurring substance abuse and mental health disorders and histories of violence	Case study/report
Mccalman [110]	2020	Australia	Children from remote indigenous communities	Healthcare and wellbeing support staff	Health	Boarding school health and wellbeing service	Examines how boarding schools across Queensland promote and manage healthcare and wellbeing support for Indigenous students.	Qualitative
Mccarthy [111]	2020	US	Social work students	Social work educators	Education	University	Explores methods that instructors can take to support students’ developmental growth through the concept of intersubjectivity within a relational theory framework.	Opinion piece/theoretical argument
Mcdonald [112]	2013	US	Trainee teachers	Teacher educators	Education	University	Examine how placements in community-based organisations enable trainee elementary school teachers to practice relationally	Qualitative
Mcmahon [113]	2011	US	Patients	Nursing professionals	Health	General nursing	Propose a mid-range theory of nursing presence, identify development opportunities to improve student nurse use of presence as a relational skill	Opinion piece/theoretical argument
Mcpherson [114]	2018	Australia	Children	Social workers, psychologists, and managers	Social care/work	Residential childcare	Reports on a study of a program response to children who have experienced trauma and are placed in out-of-home care	Mixed methods
Meer [115]	2017	South Africa	Women with intellectual disabilities	Service providers	Social care/work	Non-governmental disability service providers	Describes the intricacy of familial relationships for women with intellectual disabilities in South Africa who experience gender-based violence	Qualitative
Miller [116]	2020	UK	Adult carers	Service providers	Social care/work	Carer support	Explore practitioners’ views about the role of the narrative record in holding memories, recognition of capable agency, clarifying possibilities for action, restoration of identity and wellbeing.	Qualitative
Moore [117]	2021	UK	Service users	Adult social care services	Social care/work	Local Authority (Adult Social Care)	Using a case study of a large UK local authority adult care department, describes a new practice model, moving away from transactional practice and promoting creative, autonomous, and relationship-based practice	Case study/report
Moore [118]	2020	UK	Mental health service users	Peer support workers	Health	NHS Mental Health Services	Explore what NHS mental health professionals value about the peer support worker role	Qualitative
Motz [119]	2007	Canada	Women who abuse substances—pregnant or with children	Child welfare, substance use treatment, health and medical, and children’s service sectors	Health	Substance Misuse support service	Describing an early identification, prevention and treatment program for pregnant and parenting women who abuse substances (Breaking the Cycle)	Case study/report
Motz [120]	2019	Canada	Women who abuse substances with young children	Child welfare, substance use treatment, health and medical, and children’s service sectors	Health	Substance Misuse	Using a developmental-relational framework to understand women who abuse substances, their development and how this relates to early childhood experiences of violence in relationships	Case study/report
Mulkeen [121]	2020	Ireland	General social care service users	Social care workers	Social care/work	General social care	Discussing how concepts of care (including its relational components) are operationalised in social care workers standards	Opinion piece/theoretical argument
Munford [122]	2020	New Zealand	Children	Social workers	Social care/work	Youth services	Examine the experience of shame and recognition of vulnerable young people during transition to adulthood	Qualitative
Murphy [123]	2012	UK	Students	HE lecturers	Education	University	Arguing for a new pedagogy of higher education	Opinion piece/theoretical argument
Muusse [124]	2021	Netherlands	Service users	Community mental health team	Health	Community mental health team	Describe dilemmas related to multiple perspectives on good community mental health care, using multiple stories about Building U. We unravelled the stories as different modes of ordering care that are present in the daily discussions about the work of the CMHT.	Qualitative
Muusse [125]	2020	Italy	People with mental health conditions	Providers of mental health care and other support to people with mental health conditions	Health	Mental health in Trieste (minimal impatient provision)	Exploring good care in the context of Trieste deinstitutionalised mental health care system/services	Qualitative
Nelson [126]	2011	New Zealand	Children and families	Nurses	Health	Public health	Describe the community-based nursing service provided in Wellington for Children and Families	Case study/report
Nepustil [127]	2021	Czech Republic	People affected by addiction	Psychologists	Health	Addiction services	Describe an approach that uses a more relational perspective when working with people experiencing addiction	Opinion piece/theoretical argument
Newbury [128]	2012	Canada	Providers and recipients of community provision	Course provider	Education	Two-day continuing education course on community development	Reflection on the experience of developing and delivering a two-day continuing education course on community development, and potential of relational practice when self is understood as relationally constituted, and change is understood as an ontological and collective process.	Case study/report
Nicholson [129]	2021	UK	Offenders	Probation	Criminal justice	Probation co-operatives 'The Preston Model' OPD Pathway	Present a workable, cooperative, democratised organisational form for offender resettlement allied to alternative approaches for realising fairer local economic justice.	Case study/report
Noam [130]	2013	US	Students in an after-school club	Teachers	Education	After school care	Discuss the role of and challenges with youth development-orientated educators in after-school club provision in schools in building relationships with students.	Opinion piece/theoretical argument
Noseworthy [131]	2013	New Zealand	Women prenatal and postnatal	Midwives	Health	Midwifery	Critically explores current issues around decision-making and proposes a relational decision-making model for midwifery care.	Qualitative
O'Meara [132]	2021	UK	Offending women	Offending managers	Criminal justice	Probation	To explore women’s experiences of criminal justice systems to inform the development of guidance on working with women.	Qualitative
Ould brahim [133]	2019	Canada	Patients with chronic pain	Nursing staff	Health	Self-management of care	Review predominant critiques of self- management and the traditional individualistic view of autonomy, proposing a relational approach to autonomy	Opinion piece/theoretical argument
Pahk [134]	2021	South Korea	Solitary seniors	Peer support workers	Social care/work	Peer-support Services	Presents a relational framework for peer-support design and its application to two existing peer-support services for solitary seniors to understand the multi-faceted issue of social support	Mixed methods
Parker [135]	2002	US	Patients	Care workers	Health	Teaching hospital	Advances a model of workgroup-level factors that influence relational work, based on data from case studies of two caregiving workgroups.	Case study/report
Plamondon [136]	2018	Canada	Health care system users	Health care system providers	Health	Formal and informal providers of health care systems as well as community-based organizations	Outline the deliberative dialogue method and reflect on how these practices can help establish both processes and outcomes that can affect meaningful change in health systems	Opinion piece/theoretical argument
Porter-samuels [137]	2019	Tonga	Students	Teachers	Education	Five schools which form a Kahui Ako	Insights from a group of predominantly pakeha teachers grappling with culturally responsive relational practice (CRRP), in a time and environment where external factors can affect self-efficacy and limit personal agency.	Mixed methods
Pozzuto [138]	2009	US	Social work service users	Social workers	Social care/work	General social work	Review the literature that calls for the incorporation of relational theory into social work practice involving two strands: the psychoanalytic and the feminist	Critical/narrative review
Quinn [139]	2015	UK	Students	School leaders	Education	School context not mentioned	Evaluation of a reflective learning programme developed by educational psychologists for school leaders in exploring the implementation of compassionate, relational approaches in schools, using an integrated whole school framework	Mixed methods
Rimm-kaufman [140]	2004	Us	School children	Teachers and staff	Education	Grades kindergarten through 3	Examine the ways in which experience with a relational approach to education, the responsive classroom (RC) approach, related to teachers' beliefs, attitudes and teaching priorities	Mixed methods
Segal [141]	2013	US	Spanish-speaking immigrant clients	Social workers	Social care/work	Home visitation with immigrant clients	Applies relational theory to implementation issues around early childhood home visitation with Spanish-speaking immigrant clients	Opinion piece/theoretical argument
Smyth [142]	2007	UK, USA, Australia, Canada, New Zealand	Students	Teachers	Education	Secondary school	Present a rationale for reinserting the relational work of schools at the centre of a teacher development-led form of recovery	Opinion piece/theoretical argument
Steckley [143]	2020	UK	Children and young people in care	Residential care staff	Social care/work	Residential care (children and youth)	Identify and explore potential threshold concepts in residential childcare, with a corollary question about the utility of threshold concept theory in considering student and practitioner learning.	Qualitative
Svanemyr [144]	2014	Norway	Adolescents	Health care professionals	Health	Adolescent sexual and reproductive health	Provide a conceptual framework and the key elements for creating enabling environments for adolescent sexual and reproductive health (ASRH).	Opinion piece/theoretical argument
Swan [145]	2018	Ireland	Children and young people in care	Residential care staff	Social care/work	Residential care (children and youth)	Explores the psychodynamics of relationship-based practice from the perspective of young people in residential care.	Qualitative
Thachuk [146]	2007	Canada	Prenatal and postnatal women/birthing people	Midwives	Health	Midwifery—bioethics	Examines the parallels between the Canadian midwifery model of care and feminist reconfigurations of autonomy and choice.	Critical/narrative review
Thermane [147]	2019	South Africa	Pupils and parents	Teachers	Education	Schools in low-resourced communities	Argue for the use of the curriculum to make schools in low-resourced communities become effective despite the chronic adversities they face on a daily basis.	Opinion piece/theoretical argument
Townsend [148]	2020	UK	Residents	Big local representatives	Social care/work	Community development work	Presents findings on the potential role of money as a mechanism to enhance capabilities from an on-going evaluation of a major place-based initiative being implemented in 150 neighbourhoods	Qualitative
Trevithick [149]	2003	UK	Social work service users	Social workers	Social care/work	General social work	Discuss the importance of a relationship-based approach within social work, within a psychosocial perspective, in relation to eight areas of practice	Opinion piece/theoretical argument
Tudor [150]	2020	New Zealand	Children	School social workers	Social care/work	Post-earthquake recovery work	Outlines some findings from an inquiry undertaken in the aftermath of 2011 earthquake in Christchurch, New Zealand, in which positive critique was used to examine the practice accounts of twelve school social workers alongside characteristics of recovery policies.	Qualitative
Turney [151]	2012	UK	Involuntary clients	Social workers	Social care/work	Child protection	Focuses on the process of engaging with families where a child is at risk of harm and considers a relationship-based approach to work with ‘involuntary clients’ of child protection services.	Opinion piece/theoretical argument
Turney [152]	2001	UK	Clients of child protection services	Social workers	Social care/work	Child protection	Examines the effects of physical and emotional neglect on children and considers effective social work intervention strategies for working with them and their families, making the argument that cases of chronic neglect, all involve the breakdown or absence of a relationship of care.	Opinion piece/theoretical argument
Valaitis [153]	2018	Canada	Patients	Primary health care & public health	Health	Primary care and public health	Examine Canadian key informants’ perceptions of intrapersonal (within an individual) and interpersonal (among individuals) factors that influence successful primary care and public health collaboration	Qualitative
Veenstra [154]	2014	Canada	General public/patients	Healthcare professionals	Health	Health promotion and public health	Discusses Bourdieu’s relational theory of practice in relation to agency health promotion and public health research	Critical/narrative review
Veenstra [155]	2014	Canada	General public/patients	Healthcare professionals	Health	Health promotion and public health	Advocate for a relational approach to the structure–agency dichotomy, suggesting that relational theories can provide useful insights into how and why people ‘choose’ to engage in health-related behaviours.	Opinion piece/theoretical argument
Vielle [156]	2012	New Zealand	Maori people	People working within criminal justice	Criminal justice	Criminal justice	Examines the philosophy of justice embodied in Tikanga Mãori, the Mãori traditional mechanism and approach to doing justice which adopts a holistic and relational lens	Qualitative
Ward-griffin [157]	2012	Canada	End-of-life patients	Palliative care nurses	Health	Palliative care	Examines the provision of home-based palliative care for Canadian seniors with advanced cancer from the perspective of nurses.	Qualitative
Warner [158]	2015	US	Severe disabilities	Educators and Therapists	Education	Community-based Special Education	Addresses the importance of community in fostering transformative learning and living environments for children with special needs.	Opinion piece/theoretical argument
Webber [159]	2017	UK	Children	Teachers	Education	Primary school	Explore how the case study school defines their approach and identify the strategies they put in place to support looked after and adopted children.	Case study/report
Werder [160]	2016	US	Students	Teachers	Education	Universities	Using examples from two institutions, partnerships with students in the scholarship of teaching and learning (SOTL) category of the partnership model are explored, focussing particularly on “co-inquiry”	Case study/report
Williams [161]	2009	UK	Older people	Nursing and care staff	Health	Acute settings for older people	Argue that the care of older people in acute settings will not be improved until more emphasis is given to the nature and quality of relationships between practitioners, older people and their carers recognising the importance of ‘relational practice’ as the basis for high-quality care.	Opinion piece/theoretical argument
Williams [162]	2018	UK	Families	Family practitioners	Social care/work	Family service delivery	Describes the findings of an evaluation of a training programme; The Restorative Approaches Family Engagement Project	Mixed methods
Wortham [163]	2012	US	Teachers, students	Educational psychologist	Education	Educational psychology	Outlines the implications of Gergen’s [164] relational approach for educational research and practice.	Opinion piece/theoretical argument
Wright [165]	2012	Canada	End-of-life patients	Nurses	Health	End-of-life care	Discusses the McGill Model of Nursing [166] provides for a relational approach that is congruent with the philosophy of palliative care.	Opinion piece/theoretical argument
Wyness [167]	216	UK	Students	Teachers	Education	Secondary school	Explores social and emotional work carried out in a case study of a school in an area of considerable economic deprivation.	Qualitative
Younas [168]	2017	Pakistan	Coronary care patients	Nurses	Health	Coronary hospital care	Describe the usefulness of the relational inquiry approach by analysing a patient’s health-illness transition and the nurse-patient interaction in Pakistan	Case study/report
Younas [169]	2020	Canada	Patients	Nurses	Health	Hospital care	Describe the relational inquiry nursing approach and illustrate how this approach can enable nurses to develop a deeper awareness of patient suffering	Case study/report

^a^Country of practice/organisation or where not stated country of author/s

### Country

**Fig. 2:**
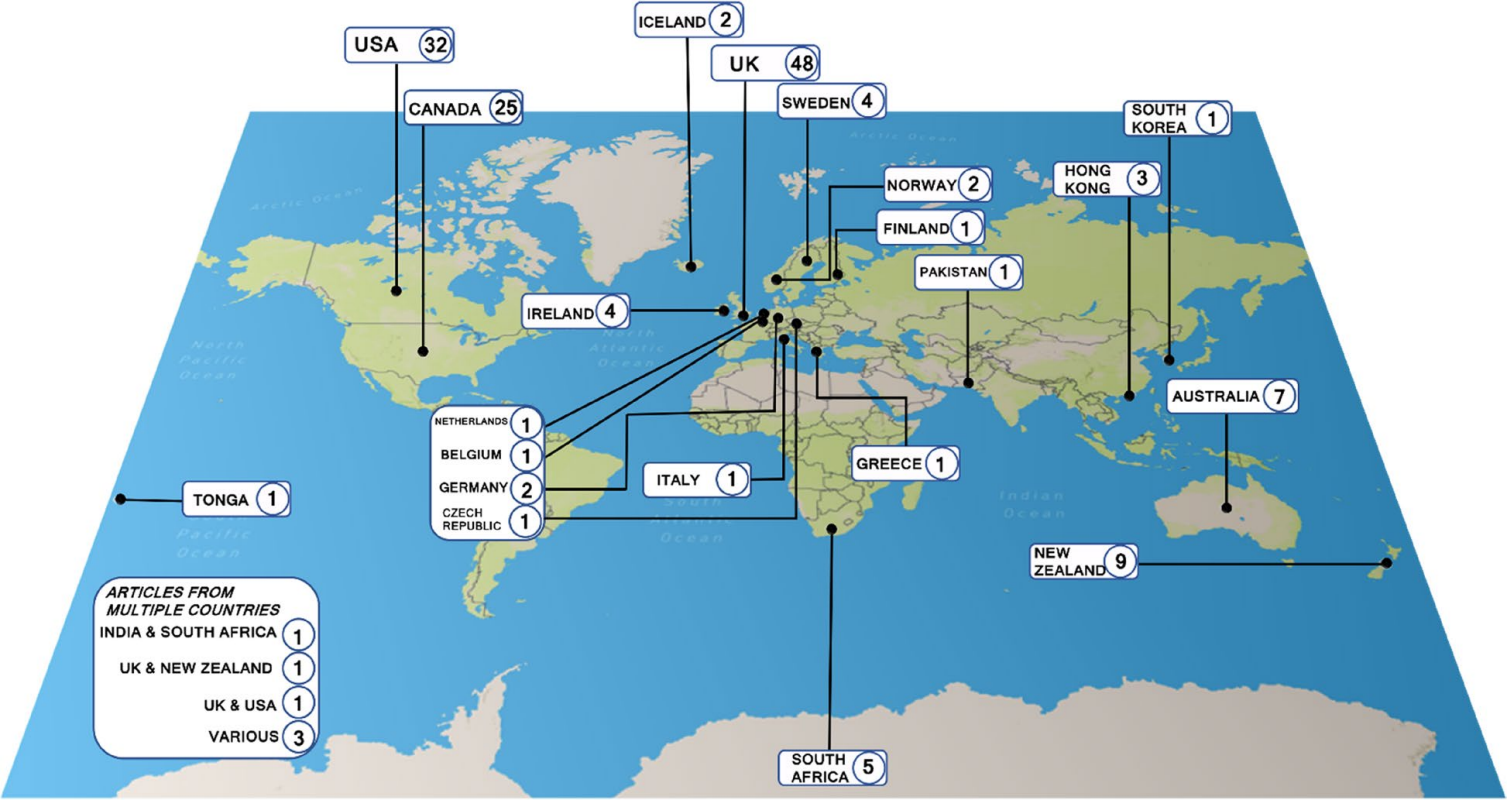
Frequencies of countries represented in the included articles/studies

Country relating to the service or organisation discussed was extracted from the included articles, where this was not identified the author’s affiliated country was noted. Figure 2 displays the countries represented in the included articles. Most of the included articles were from the UK (*n* = 48, 30.38%), followed by the USA (*n* = 32, 20.25%) and Canada (*n* = 25, 15.82%). There was however a broad and global spread of literature across the rest of the included literature.

### Sector type

In relation to the four sector types, health has the highest proportion of included literature (*n* = 60, 37.92%), followed by education (*n* = 41, 25.95%), social work/care (*n* = 39, 24.68%), and criminal justice (*n* = 17, 10.76%), with 1 paper that was across the 4 sectors [3]. Table 3 displays the frequencies of the sector types across the included articles/studies alongside the specific service/focus within each sector.

**Table 3:** Frequencies of articles/studies for each sector type with specific service type or focus

Sector type	*n* (%)	Articles/studies
**Health**	**60 (37.97%)**	
*Mental health*^a^	12 (20.00%)	[2, 11, 16, 19, 20, 43, 46, 85, 101, 118, 124, 125]
*Recovery and substance abuse*	7 (11.67%)	[14, 47, 72, 109, 119, 120, 127]
*Elderly residential/inpatient care*	7 (11.67%)	[12, 24, 31, 33, 49, 64, 82]
*General nursing/healthcare systems*	8 (13.33%)	[59, 62, 67, 100, 113, 135, 136, 169]
*Primary care/public health/health promotion*	4 (6.67%)	[126, 153–155]
*Acute hospital care*	3 (5.00%)	[32, 60, 161]
*Emergency care*	3 (5.00%)	[68–70]
*Midwifery*	2 (3.33%)	[58, 131]
*Bioethics*	2 (3.33%)	[88, 146]
*Palliative/end-of-life care*	2 (3.33%)	[157, 165]
*School nursing/health projects*	2 (3.33%)	[107, 110]
*Family nursing*	2 (3.33%)	[14, 36]
*Other nursing/healthcare*^b^	6 (10.00%)	[39, 63, 92, 133, 144, 168]
**Education**	**41 (25.95%)**	
*Compulsory education school context*	9 (21.95%)	[30, 44, 54, 79, 83, 104, 105, 108, 139]
*University*	7 (17.07%)	[29, 40, 73, 111, 112, 123, 160]
*Secondary school*	5 (12.20%)	[41, 65, 75, 142, 167]
*Kindergarten/elementary/primary school*	4 (9.76%)	[94, 98, 140, 159]
*Specialist schools*^b^	3 (7.32%)	[90, 91, 137]
*Nursing education*	2 (4.88%)	[61, 81]
*Social work education*	2 (4.88%)	[23, 53]
*After-school care/education*	2 (4.88%)	[97, 130]
*Practitioner CPD short courses*	2 (4.88%)	[80, 128]
*Early years education training*	1 (2.44%)	[45]
*Multi-cultural urban schools*	1 (2.44%)	[35]
*schools in low-resourced communities*	1 (2.44%)	[147]
*Community-based special education*	1 (2.44%)	[158]
*Educational psychology*	1 (2.44%)	[163]
**Social work/care**	**39 (24.68%)**	
*Child protection*	6 (15.38%)	[27, 71, 93, 102, 151, 152]
*Residential childcare*	5 (12.82%)	[34, 38, 143, 145, 170]
*General social work*	5 (12.82%)	[17, 42, 76, 103, 138]
*Community development work/projects*	5 (12.83%)	[48, 86, 89, 148, 149]
*Children and youth services*	4 (10.26%)	[55, 77, 78, 122]
*Children and families*	3 (7.69%)	[26, 84, 162]
*Disability social care services*	2 (5.13%	[87, 115]
*Migration/asylum seekers*	2 (5.13%)	[99, 141]
*General social care*	2 (5.13%)	[118, 121]
*Other social care*^b^	5 (12.83%)	[95, 96, 116, 134, 150]
**Criminal justice**	**17 (10.76%)**	
*Young offenders*	5 (29.42%)	[15, 25, 37, 50, 52]
*Probation/prison/re-entry to society*	6 (35.29%)	[21, 22, 28, 57, 129, 132]
*Female offenders*	2 (11.65%)	[18, 74]
*Hard to engage young people*	1 (5.88%)	[51]
*Policing*	1 (5.88%)	[66]
*International peace building*	1 (5.88%)	[106]
*General criminal justice*	1 (5.88%)	[156]

Older persons residential care has been included in the Health sector category as this care is conducted by both health and social care organisations but is with a population with predominately healthcare needs

^a^only one of the mental health studies in the health sector was with children and adolescents (the rest were adult studies)

^b^Other nursing/healthcare includes a study each on dementia, dialysis, coronary, AIDS, pain management, adolescent sexual and reproductive health, Specialist schools include one study each for schools for children with emotional, behavioural and educational issues, public charter schools, five schools forming a Kahui Ako, Other social care includes post-earthquake recovery work, peer support services, carer support, veteran reintegration support, domestic violence

### Study/article type

Study/article types were categorised as scoping or systematic reviews, empirical studies (either qualitative, quantitative, mixed methods), case studies, critical/narrative or opinion pieces/theoretical arguments. There was some overlap between the last three categories (e.g., some publications emphasising opinion or theoretical argument may also have included some brief details about application in a specific site/s). Where there was overlap the coders recorded the category that was closest to the authors’ stated intention (i.e., the aims of the article).

Descriptions of these articles were as follows:

- *Case study*—a discussion or examination of relational practice or application of a delivery model in a specific site/s.
- *Critical/narrative review*—generalised critique of relational practice or a particular philosophical approach and/or critical review of literature
- *Opinion piece/theoretical argument*—where authors are proposing a particular approach or theoretical model for relational practice and/or an opinion of how to apply relational practice in a specific sector or the importance of relational practice in a specific sector

A large proportion of publications were opinion-based or theoretical argument papers (*n* = 61, 38.60%), with a further 6 (3.80%) critical or narrative reviews. These publications tended to propose models of relational practice or make arguments for the use of the relational practice in a specific service or organisation (see Table 2 for a summary of the aims of these studies/articles). An additional large number of included publications reported qualitative studies (*n* = 45, 28.48%) or case studies (*n* = 27, 17.09%). There was a lack of effectiveness trials or quantitative studies, with only 6 (3.80%) using a quantitative design and 9 (5.70%) using a mixed methods design.

Of the included publications, 27 were categorised as case studies, and 16 were descriptions of a practice or organisation and did not include any empirical data, although some did provide literature review and/or citations of other evidence sources relating to effectiveness [21, 101]. In these publications, the focus was predominately on explaining the relational practice being used in an organisation or a specific intervention and its components, rather than conducting an evaluation or examination of the effectiveness of the service. Four of these case studies were related to criminal justice services; one discussed the application of democratic therapeutic communities in the prison service [21], a further two discussed transition programmes for female offenders [28, 74] and one a community wealth-building project to promote successful re-entry for people released from prison [129]. Five of these articles related to an education setting and discussed applying the relational practice in the provision of faculty support [73], mentoring [108], delivery of a CPD course in community development [128], approach in a special school for adolescents with emotional, behavioural and educational issues [90] and using co-inquiry to form a partnership between students and lecturers in a university [160]. Six studies were health-related describing interventions for women with substance use problems and mental illness [72, 119], community-based nursing service for children and families [126] and a school obesity programme [107]. A final case study of a large UK local authority adult care department to describe a new practice model for social care [118].

The other 11 case studies included numerically informed quantitative data evaluating the service, intervention or practice described and are included alongside the studies using qualitative, quantitative or mixed-method study designs in Table 4. Most of the empirical evidence relating to the case studies is qualitative and focussed on the perceived impacts and benefits of the provision/intervention (see Table 4). Findings also relate to the importance of an institutional buy-in to the practice and support and training of staff in the approach to ensure success [41, 57, 135].

**Table 4:** Descriptions of empirical studies (qualitative, quantitative and mixed methods, including case studies with empirical data)

Author	Date	Description	Participant characteristics	Sample size	Data collection	Data analysis	Key findings relating to relational practice
*Case study/report with empirical data*							
Berzoff [23]	2006	Describes and evaluates a master’s programme for social workers	Social workers working an average of 15.3 years in end-of-life care; ages from 26 to 71, Mage = 47.7 years; range of settings, including hospitals, community-based agencies, prisons, hospices, and nursing homes.	Not mentioned	Secondary data analysis of student’s evaluations	Thematic analysis	The hours students spent with their supervisors modelled how to be fully present with their dying and bereaved clients
Campbell [39]	2012	Secondary analysis of two contrasting case studies using peer education as the starting point for involving highly marginalised women in HIV/AIDS management.	Women with AIDS living in the community	Not mentioned	Secondary data analysis of case studies	Case comparison	Transformative communication (TC) flourished in one site and failed in another; TC is unlikely to be practiced in a non-transformative context, aspects of the material, symbolic and relational contexts of the two case studies profoundly shaped the possibility of successful TC. Creating enabling environments for transformative communication is a crucially important, though often neglected element of community health programmes.
Celik [41]	2021	Investigate how a secondary school improves the educational engagement of working-class, second-generation Turkish immigrant youth in Germany.	Students aged 15–18 years	14 students and 10 teachers	Semi-structured interviews, observation, documentary analysis	Qualitative content analysis	The school’s institutional habitus combines the communal values of the immigrant community and the middle-class academic practices; the former narrows the gap between home and school, and the latter modifies the classed feelings of students.
Davies [57]	2019	Examines the progress of introducing Enabling Environments (EE) standards across seven sites	Approved premises residents and prison inmates	Four approved premises (24–26 residents) 3 prisons (250–1000 inmates)	Part of a larger EE impact study—observations/discussion with staff, service feedback, prison resident responses	Thematic analysis	It is essential for those leading services and new initiatives to engage with staff on the ground to demonstrate why the change is necessary and should be pursued now. Those involved in service development need to have sufficient knowledge and understanding to make links between their practice and the standards/goals and need to be able to “buy into” the process. While Leadership and Involvement are two of the 10 EE standards, it appears that these might be considered foundation areas which are required as a platform onto which the other aspects of the process “sit.”
Durocher [64]	2019	To discern relational approaches adopted by families in planning for the discharge of older adults from inpatient settings	Patients, family members and professionals involved in discharge meetings (family conferences) in three case studies	*N* = 20 (five older adults, seven family members and eight healthcare professionals)	Secondary analysis of micro ethnographic case studies, including observational field notes & semi-structured interviews	Qualitative exploratory	Family members employed strategies to promote older adults’ participation in decision-making that were consistent with relational autonomy theory, to overcome tensions between older adults’ wishes to return home and family’ assumption of a primary role in discharge decision-making and their wish for the older adult to move to a supported setting
Kitchen [94]	2009	Examines how a respectful and relational approach to teacher development can result in deep and sustained professional growth and renewal.	One participant/researcher	1	Field notes and journals, reflections, and teaching documents	Narrative inquiry	Relational teacher development affirms the centrality of relationship in professional development and renewal.
Markoff [109]	2005	Describes the “relational systems change” model developed by the Institute for Health and Recovery, and implementation to support integrated and trauma-informed services for women with co-occurring substance abuse and mental health disorders and histories of violence	Service providers; women clients; key staff	9 focus groups, number of interviewees not mentioned,	Focus groups, Semi-structured interviews	Qualitative analysis, network analysis	The WELL Project demonstrates that a highly collaborative, inclusive, and facilitated change process can affect services integration within agencies (intra-agency), strengthen integration within a regional network of agencies (interagency), and foster state support for services integration.
Parker [135]	2002	Describes a model of workgroup-level factors that influence relational work, based on data from case studies of two caregiving workgroups.	Two women’s primary health care groups, each situated in a teaching hospital in a large city, one site was part of a private, university-owned hospital while the other was part of the nationwide Veterans Affairs (VA) health system.	Not mentioned	Documentary analysis and observations of meetings	Constant comparative method	Relational work in caregiving organizations depends not only on the skill of individual providers and care seekers but also on the extent to which the work group and organization are structured and operate in ways that are supportive of relational work behaviours. The more that such groups are conscious of themselves as groups that serve important functions in supporting the work of their members, the more likely they will be able to fill this role
Webber [159]	2017	Explore how the case study school defines their approach and identify the strategies they put in place to support looked after and adopted children.	Head Teacher, intervention team leader/senco/Designated Teacher, Previous Class Teacher–Year 1, Previous Class Teacher–Year 2, Current Class Teacher–Year 3, Current Teaching Assistant	6	Semi-structured interviews	Thematic framework analysis	The approach involves six main components: Whole school approach of a therapeutic PACE attitude, Communication between staff including support for transitions, Physical contact–touch, regulating emotions, Bespoke provision for each child, Not shaming children, Working with families and multi-agencies
Younas [168]	2017	Describe the usefulness of the relational inquiry approach by analysing a patient’s health-illness transition and the nurse-patient interaction.	1 case study: a 43-year-old patient, was admitted through the emergency department to the coronary care unit (CCU) of a private hospital in Islamabad, Pakistan, with chief complaints of severe chest pain, diaphoresis, and shortness of breath.	1	1 case exemplar	Relational inquiry approach	The relational inquiry approach can help nurses to look beyond superficial clinical situations and recognize the impact of various contexts in nursing practice. It helps nurses to recognize the fact that they should not disregard the factors influencing the clinical situations but focus on their precise features. The use of the relational inquiry approach can help them to understand the diversity in the attitudes, beliefs, and values of people and enables nurses to engage in an authentic and respectful nursing inquiry to improve the nursing care given to a particular patient.
Younas [169]	2020	Describe the relational inquiry nursing approach	2 case studies: 1 male, a 43-year-old man admitted with non-ST elevation myocardial infarction. 1 female, an 81-year-old woman who was admitted to the hospital after a fall in a bathtub	2	2 case exemplars	Relational inquiry approach	The relational inquiry approach could play an essential role in developing an effective relationship with the patient and the family and explore their suffering at a deeper level. The nurses were able to recognize the complex interplay of intrapersonal, interpersonal, and con-textual factors.
*Mixed methods*							
Blagg [25]	2018	Reports on a study undertaken in three Indigenous communities in the West Kimberley region of Western Australia (WA) intended to develop diversionary strategies for young people with fetal alcohol spectrum disorder (FASD).	Representatives from key mainstream agencies (police, health, justice, education); Indigenous service providers and youth agencies.	122	Interviews and focus groups	Thematic analysis	‘decolonising’ approach; support for ‘community owned’ rather than ‘community based’ diversionary options. Mobile ‘needs focused’ court could draw on the techniques employed by ‘problem-oriented courts’ to promote better outcomes for young people with FASD.
Elliott [66]	2011	Test a relational model of authority in victim-police interactions.	People who had reported a crime (personal or property) to the police in the previous year	110	Semi-structured interviews, quant data on victim demographics + type of crime reported/self-report measures of procedural justice, legitimacy and justice sensitivity and Social Desirability	Grounded theory analysis; constant comparison method; correlational and hierarchical regression analysis	The quantitative results supported the hypotheses that higher perceived antecedents of procedural justice would be associated with higher perceived legitimacy, outcome fairness, and satisfaction with the contact. Antecedents of procedural justice were a stronger predictor of outcome fairness and satisfaction than the realization of a desired outcome, and a stronger predictor of legitimacy than criminal history. Qualitative findings supported these results.
Ferguson [71]	2020	To examine what social workers actually do, especially in long-term relationships	Social work teams, families in contact with these teams	Two differently configured SW teams	Ethnography including observational field notes & semi-structured interviews	Thematic analysis and constant comparison	Findings show that social work some of the time has a significant amount of involvement with some service users and the dominant view that relationship-based practice is rarely achieved is in need of some revision. Drawing on relational, systemic, and complexity theories, the paper shows how the nature of what social workers do and culture of practice are shaped by the interaction between available services, office designs, and practitioners', managers', and service users' experiences of relating together
Mcpherson [114]	2018	Reports on a study of a program response to children who have experienced trauma and are placed in out-of-home care.	Graduated young adults, carers, social workers, psychologists, and manager	3 interviews (young adults), carer focus group and 14 interviews (key professionals), 48 client files (children)	Interviews, focus group, analysis of client files	Narrative inquiry	Key findings highlight the significance of relational practice to interrupt the projected trauma trajectory and for young people to stabilise and self-regulate
Pahk [134]	2021	Presents a relational framework for peer-support design and its application to two existing peer-support services for solitary seniors in Seoul and Ulsan to understand the multi-faceted issue of social support	All female, aged between 65 to79	14	Case studies using observation and interviews and quantitative data	Content analysis, network analysis (peer support)	Peer-support services can be better targeted to meet the relational needs of peers, and thus offer a relational approach to analysing and designing peer-support services. This relational approach employs a set of metrics that correspond to the multifaceted characteristics of a support network.
Porter-Samuels [137]	2019	Insights from a group of predominantly Pakeha teachers experiences of culturally responsive relational practice (CRRP)	Survey respondents’ professions: classroom teacher – *N* = 33; Teacher-aide – *N* = 5; SENCO – × 2; and ‘Walking’ school leader – *N* = 6). Focus groups info: Pakeha (*N* = 12), female (*N* = 13), and experienced teachers (< 5 years × 1; 5–10 years N = 5; > 10 years *N* = 10)	46 (survey), 14 (focus groups)	Anonymous survey & Focus Groups	Inductive thematic analysis	Five distinct themes: the centrality of relationships of care, owning one’s learning journey, contextual influences and environment, the preciousness of time, identity and wellbeing
Quinn [139]	2021	Evaluation of a reflective learning programme developed by educational psychologists for school leaders in exploring the implementation of compassionate, relational approaches in schools, using an integrated whole school framework.	School leaders	44 school leaders from 32 schools participating in programme	Reflections on achievements using a Likert scale, world café method (12 weeks later)	Mean ratings compared by school, narrative synthesis	Whole school approaches have been identified as central to the strategy ‘Transforming children and young people’s mental health provision’, with reference to whole school thinking as multi-component, coordinated and coherent rather than piecemeal interventions
Rimm-Kaufman [140]	2004	Examine the ways in which experience with a relational approach to education (responsive classroom (RC) approach) related to teachers’ beliefs, attitudes, and teaching priorities	69 teachers in grades kindergarten through 3 at 6 schools (3 schools in their first year of RC implementation and 3 comparison schools)	140 Three schools implementing the RC approach and three comparison schools	Questionnaire and Q sort exercise	Regression analysis	Teachers who reported using more RC practices reported greater self-efficacy beliefs and teaching practice priorities that were consistent with those of the RC approach. Teachers at RC schools were also more likely to report positive attitudes toward teaching as a profession and to hold disciplinary and teaching practice priorities that were aligned with the goals of the RC approach.
Williams [162]	2018	Evaluation of a training programme (The Restorative Approaches Family Engagement Project) delivered to voluntary sector family practitioners across Wales to increase used of RA	Questionnaire: 78% female, 20% male. Age ranged from under 30 to over 60 years old. All participants worked within the social care sector, most in family work and support roles (40 out of 81 respondents), housing (*n* = 13), and domestic abuse work (*n* = 8). Focus groups: 78% were female and 22% male, worked in housing associations/support (*n* = 10); family support (*n* = 5); mental health ser-vices (*n* = 3); domestic abuse support (*n* = 2); youth work (*n* = 2); and cancer support (*n* = 1)	112 (questionnaire) 23 (focus groups)	Questionnaire & focus groups	Deductive thematic analysis (focus group data). Statistical analysis (questionnaire)	Increases in practitioner confidence in using RA support wider evidence that links skills development with increased self-efficacy and confidence [171–174]. Moreover, the increases in all aspects measured: relationships with service users, communication, identifying user needs and goals, and facilitating change, give a rationale for why practitioners felt they could engage and work with families and clients better post-training; as well as indicating that RA gave their practice a necessary framework and set of tools that had a positive effect on the whole process of service provision and was more likely to engage service users, and stimulate changes.
*Qualitative*							
Anderson [12]	2016	Exploring the complexities of care; working environments; and knowledge, skills, and efforts of care aides who work in nursing homes.	Care aides	22 (2 in private nursing homes, 20 in publicly funded homes)	Interviews	Inductive interpretive analysis	Four themes were identified that contributed to an overall understanding of participants’ caring practices and relationships with residents and families: (a) Desiring the Ideal Relationship, (b) Establishing Relationships with Residents and Their Families, (c) Maintaining Relationships with Residents and Their Families, and (d) the Reality of Care Aide Work.
Bainbridge [18]	2017	Considers the development of the therapeutic environment of a (Psychologically Informed planning Environment) PIPE Unit and in particular its translation for women in custody	Female offenders	Not stated	Narratives from focus groups	Thematic analysis	Importance of the environment; importance of relationships with staff; Importance of ordinary activities; Importance of the community
Bjornsdottir [24]	2018	Enhance knowledge and understanding of the nature of home care nursing practice.	Members of five home care nursing teams and 15 older persons receiving care at home	Elderly care receivers n = 15; team leader nurses n = 8	Observations; interviews	Thematic analysis	Making a 'net' around each patient; organisational working "translational mobilisation
Blumhardt [26]	2017	Explores the radical practice of anti-poverty organisation ATD Fourth World in England (where child protection is characteristically risk-averse, individualised, and coercive), as an alternative for work with families experiencing poverty and social exclusion	Trainee social workers	5	Transcribed interviews, discussion	Non-specific thematic/narrative analysis of qualitative data	Constructing and bolstering relationships… Reflecting a “relational welfare” approach that eschews the isolating, “transactional” approach of neoliberal social services to promote meaningful relationships within families and communities, and between social workers and families. Understanding born of shared experience is key
Bøe [27]	2019	Examine factors described by milieu therapists as significant for relational work with youth placed in institutions	Four milieu therapists working in child protection institutions	4	Transcribed interviews	Interpretative phenomenological analysis	1 Structural and personal factors are both important for relational work. 2 Relational work is based in everyday events. 3 Time and togetherness create opportunities for shared understanding of the youth’s resources. 4 The potential in ‘togetherness’ reduces asymmetry and promote an equal relationship. 5 The milieu therapist’s ability to move between closeness and distance to the youth is crucial for the relationship.
Bridges [32]	2017	Identify and explain the extent to which Creating Learning Environments for Compassionate Care (CLECC) was implemented into existing work practices by nursing staff, and to inform conclusions about how such interventions can be optimised to support compassionate care in acute settings.	Nursing staff, ward managers	47	Staff interviews (*n* = 47), observations (*n* = 7 over 26 h) and ward manager questionnaires on staffing (*n* = 4).	Narrative synthesis—using normalisation process theory as a framework	Relational work in caregiving organisations depends on individual caregiver agency and on whether this work is adequately supported by resources, norms and relationships located in the wider system. The success of the intervention was dependent on coherence and understanding of the principles—which generally reflected their philosophy for compassionate care. Staff were keen to participate but were not always clear whose responsibility it was to drive things forward. Reflective monitoring was valued by staff but difficult to sustain.
Brown [34]	2018	Explores the views and experiences of 26 residential care workers in the Republic of Ireland regarding relationship‐based practice	Residential care home workers	26	Semi-structured interviews	Thematic analysis	Relationship‐based practice has not been fully understood and/or embraced in practice because of a culture of fear that has permeated the Irish residential childcare system. Using theoretical concepts associated with the sociology of fear, the paper explores their effects on practice and argues that these are amplified given the current low status of residential care workers, the impact of media reports and the influence of current discourses around professional practice in which ‘objective’ and ‘emotionally detached’ practice is viewed as synonymous with efficiency and effectiveness.
Cahill [38]	2016	Exploring relationship-based approaches in residential childcare practice, from the perspectives of both residential childcare workers and young care leavers	Professionals and care leavers	32 professional and 4 care leavers	Focus groups and interviews	Not mentioned	Relationship based approach in residential care practice is grounded in the knowledge and skill base of the care worker along with characteristics and circumstances of both the care worker and the young person. Although these elements were identified as important for enhancing relationship-based practice, they were also considered factors that can mitigate against the quality of relationships formed. The ability to achieve relationship-based practice in residential childcare units rely heavily on both the young person's background and circumstances and the personality of the staff and their capacity to positively engage with such individuals.
Carpenter [40]	2015	Examine the strategic organization-public dialogic communication practices of universities in the USA	Campus sustainability and student group leaders, 12 males and 25 females: professionals-41.62 years old and students- 23.29 years old.	37	Semi-structured interviews	Thematic analysis	The dialogic model of communication provides some unique insight into how IHE are positively cultivating a culture of sustainability on campuses by using communication to bring about mutually beneficial relationships. The results also reveal that SL most likely engage in empathy, followed by propinquity, mutuality, commitment and risk with students and staff as their main focus of their communication efforts.
Conradson [48]	2003	Explores the ways in which drop-in centres may at times function as spaces of care in the city.	Service users, 2 senior managers interviewed and 8 volunteers	4 months of observation + two interviews with senior staff and questionnaires with 8 volunteers	Observations; interviews	Narrative synthesis—Carl Rogers’ [175] core conditions for successful therapeutic encounter used as framework	The emergence and endurance of such spaces depends both upon the willingness of some individuals to move towards others and, amongst those being engaged in this way, upon a receptivity to such initiatives. Spaces of care are shared accomplishments and, in reflection of this, may at times be socially fragile.
Cranley [49]	2019	Explore shared decision-making among residents, families, and staff to identify relevant strategies to support shared decision-making in Long Term Care	Residents 70 years or older mild-moderate cognitive impairment; Relatives who visited at least monthly or where substitute decision makers for relatives	3 staff, 3 residents, 3 family members	Interviews (Individual)	Content and thematic analysis	Four main themes (and their sub-themes) that described resident, family and staff perspectives of shared decision- making: (a) oral communication pathways for information sharing (informal, indirect and formal communication pathways); (b) supporting resident decision-making autonomy (types of decisions made); (c) relational aspects of care facilitate shared decision-making (building trust and team collaboration); and (d) lack of effective communication creates barriers to shared decision-making (differing perspectives and reactive communication).
Creaney [52]	2020	Explore young people’s experiences of youth justice supervision with particular reference to the efficacy of participatory practices	Front-line professionals, operational managers, and children under youth justice supervision.	14 Front-line professionals 6 operational managers, 20 children	In-depth interview and participant observations	Thematic Analysis—Braun and Clark’s (2006) framework	Several young people were seeking to exert minimal energy to achieve a type of passive compliance with court order requirements, adopting a ‘‘ready-to-conform’’ mindset. Professionals were concerned that they were also participating in this type of ‘‘game playing’’.
Deery [58]	2008	Examines community midwives’ experience of linear time during the third phase of a 3-year action research study to compare and contrast the ways in which they experienced this temporal framework, individually and organizationally, in their clinical practice.	Community-based midwives	8	In depth interviews	Analysis using the voice centred relational method of data analysis	Relational time was less dominant and required generous, less predictable, and more responsive spending of time, which was seen as well invested in trusting relationships, client, and job satisfaction, and good clinical outcomes. Time out of client care then became a sacrifice, as the midwives viewed the researcher as encroaching on their time, which was needed elsewhere with clients, in order to focus on their own and their clients’ needs. Organizational pressures thus affect midwives’ conceptualization of time, where there is an emphasis placed on being ‘on time’ for the sake of the organization rather than ‘spending time’ with clients.
Dewar [60]	2013	Actively involve older people, staff, and relatives in agreeing a definition of compassionate relationship-centred care and identify strategies to promote such care in acute hospital settings for older people.	Registered nurses, nonregistered care staff, allied health care professionals and medical staff (*n* = 35, i.e., 85% of staff), patients (*n* = 10) and families (*n* = 12)	Medical Staff (*n* = 35) Patients (*n* = 10) Families (*n* = 12)	Observations, informal discussion, interviews, group interviews	Immersion crystallization [176]	Engaging in ‘appreciative caring conversations’ promotes compassionate, relationship-centred care but that these conversations involve practitioners taking risks. Such ‘relational practices’ must therefore be valued and accorded status. Staff require appropriate support, facilitation, and strong leadership if these practices are to flourish.
Ellery [65]	2010	Discover how RTLB (Resource Teachers: Learning and Behaviour) can effectively support secondary teachers to enhance inclusive classroom practices by investigating students' perceptions of their personal experience and perception of inclusive practices at secondary school.	Secondary school children years 7–11	4	Focus groups, photovoice	Grounded analysis/appreciative inquiry	Three themes identified: Students said that they wanted their teachers to make them feel welcome in their learning spaces and to give them a sense of value and belonging. Secondly, students said that the development of positive and respectful relationships between students and their teachers was important, involving a reciprocation of information between themselves and teachers. Thirdly, students expanded on the topic of learning needs and interests to express their ideas about how they wanted to receive learning support.
Emmamally [69]	2020	Describe Health Care Providers’ perceptions of relational practice with families in emergency department contexts.	Emergency department professionals	9	Semi-structured interviews	Content analysis	Four categories emerged from the content analysis: families and hcps connecting; recognising the uniqueness of families; caring interactions; and taking charge when necessary. Families and healthcare providers connecting: Participants likened relational practice to building a bridge that connects hcps with families, in which either party could easily reach out to the other.
Emmamally [70]	2020	Describe families’ perceptions of relational practice when interacting with health care professionals in emergency departments in the South African context.	Families of patients admitted to ED	6	Semi-structured interviews	Qualitative content analysis	Four major categories identified from family members’ perceptions: Disrupted worlds; Care is what you see and hear; Powerlessness; Feeling disconnected. The disrupted world’s theme sets context and does not speak of relational practice. Care is what you see and hear: Caring relationally involves compassionate, interdependent relationships that are characterised by connecting with people. The last two themes were about negative experiences for family members; not RP or absence of RP.
Hibbin [83]	2019	Explores a relational approach in school within the context of Nurture Groups, Restorative Practice and positive language and communication	Senior leaders, mainstream class teachers and NG specialists in seven schools in the Northwest of England for the nurture group study	14	Semi-structured interviews and observations	Constructivist grounded theory	To enact a principle of inclusion for troubled children, we need to create facilitating environments in school that are consistent, equitable and that promote trust, through naturalistic opportunities for positive language and communication.
Howitt [86]	2020	Exploration of the transformative potential of relational, rather than transactional, community development practices	Residents, local and regional members of The Salvation Army, and other local stakeholders.	52	Interviews	Thematic analysis	Extending hospitality, recognizing strangers, and building relationships were central to both the theological underpinnings and personal and institutional practice in the No. 47 Project
Ingolfsdottir [87]	2021	Views and experiences of professionals providing specialised services to disabled children and their families.	Professionals (language and speech therapy, occupational therapy, physiotherapy, preschool special education, and social pedagogy), 12 females and one male, work experience ranged from six years to about 40.	13	Focus groups	Qualitative content analysis	Discrepancies between the policy aims and the conditions for service provision. These inconsistencies affect the work situation of the professionals, who are not encouraged by their employers to work in a family-centred inclusive manner. Instead, they meet various obstacles if they strive to adapt to the wishes and needs of the families to provide services at the child’s preschool or home.
Kerstetter [91]	2016	Examines the extent to which authoritarian discipline systems are necessary for success at “no excuses” schools	10 teachers and other school staff members, all female, two participants identified as black, one identified as Latina, and the remainder identified as white, most participants under the age of 35 and two between the ages of 35 and 49.	10	Semi-structured Interviews and observation of teachers' work	Qualitative analysis	Relational approach to discipline cultivates non-cognitive skills more closely aligned with the evaluative standards of middle-class institutions, such as skills in self-expression, self-regulation, problem-solving, and conflict resolution. A comparison of academic achievement data also suggests that “no excuses” schools may be able to implement relational discipline approaches without sacrificing academic success on a key predictor of future academic performance.
Kippist [92]	2020	Findings from the first of a two-part study exploring user experiences of brilliant renal care within the Regional Dialysis Centre in Blacktown (RDC-B).	The first world café involved 28 patients (*n* = 18) and carers (*n* = 10); while the second involved 18 staff members.	46 patients, carers, and staff members	World café	Constant comparative method	RDC-B is completely patient and relationally centred, with high-quality connections, dedicated and competent staff providing a complete, responsive, and personalized service that is also like being in a family. Drawing on POS, we suggest that relational-centred care requires at the very least high-quality connections and relational coordination to build and sustain the levels of positivity identified in the RDC-B.
Kirk [93]	2016	Describes and evaluates an approach to social work practice, which divides levels of risk within the child in need category enabling adequate, coordinated support and oversight to be provided for children and families with complex needs.	Social workers (*N* = 13) and team managers (*N* = 3), three members of the Local Authority Safeguarding Board (LSCB) and two parents	16	Interviews	Interpretive discourse analysis	Practitioners and managers know that relationships lie at the heart of social work practice and that working in a relational way improves outcomes for children. They know too that an either-or approach means some children with complex needs may languish in the child in need category until a crisis moves them above the CP threshold. However, stepping outside formal procedures and processes leaves them vulnerable to criticism should things go wrong
Kong [95]	2020	To provide insights for improving the local domestic violence service, whose main focus is on crisis intervention.	All women participants had experienced both physical and psychological abuse for at least 5 years, while most of them except NF and the researcher-participant, had children aged between 12 and 17 when the inquiry began. Among women participants, one was undergoing divorce proceedings and two were still fighting for custody during the inquiry.	7	Conversations, observational data, and interactive data	Constant comparative analysis (Glaser 1978)	The relational approach emerging from the project is about acknowledging the fluidity and multiplicity of identities performed by abused women at different times and space. It is to see identity work a crucial practice for bringing women’s marginalised stories to the surface and re-organising social relationships in ways to address power differences, such as that between sympathizes and sympathizers, dependents and independents and victims and survivors.
Larkin [99]	2020	Through a study of how UYFS and practitioners in England experienced and constructed each other during their everyday practice encounters, the potential of the practice space for creating mutual understandings and enabling positive changes is discussed	Social workers, UYF asylum seekers	5 social workers; 3 UYFS	Interviews accompanied by free drawings	Analysis guided by the feminist model of Voice-Centred Relational analysis;	Rather than practice encounters being the site where policy and procedure are simply acted out, the data show them to be interpretations within unique events, ‘a locus of the generation of new trajectories and new configurations’ They can, therefore, be spaces of creativity and change, as well as relational spaces which exclude or silence.
Lefevre [102]	2019	Analysis of data from a 2-year evaluation of the piloting of a child-centred framework for addressing child sexual exploitation (CSE) in England to illuminate dilemma between control and participation, and strategies used to address it	Professional and young people	Interviews—28 professionals, 17 young people, surveys × 300, 19 observations of meetings	Interviews with professionals, observations of meetings, 2 × surveys	Thematic analysis	Strategies for reconciling protection and participation—Clarity around threshold of concern,—Rights-based ethical position,—Doing with and for: participation as ‘doing with’ young people, involving young people as partners in evaluating the riskiness of their situations. Other participants spoke of ‘doing for’. This might involve ‘holding on’ to concerns about risk for as long as it took, waiting for the young person, who might have initially rejected this view, to ‘catch-up’. It could also mean ‘being the voice’ for the young person, advocating to the professional system on their behalf to ensure that their right to autonomy, choice and privacy was considered.
Lindqvist [104]	2014	Examine the strategies used by teachers whose practice was considered inclusive (inclusive from a relational perspective)	Head teachers	5	Semi-structured interviews	Thematic framework analysis	7 themes that describe strategies of headteachers who operated a relational approach to inclusion of children with SEN in their schools: Organising work (having specific staff or time allocated to children with SEN, understanding needs to tailor support); valued solutions (flexible solutions from a range of options within the remit of teaching, personalised to student); Leadership (motivating, communicating, empowering to find solutions, participating in pedagogy, evaluating teachers, staff relationships); support staff (having teaching assistants and SENCOS—operational development staff to support teachers, as well as sufficient teachers and smaller class sizes) definitions of inclusion (varied but generally about including children with SEN in a classroom) striving for consensus (consistent understandings) welcoming outer steering (useful to have high standards and be accountable, following up students outcomes)
Mccalman [110]	2020	Examines how boarding schools across Queensland promote and manage healthcare and wellbeing support for Indigenous students.	School staff	9	Semi-structured interview	Constructivist grounded theory	Main process through which boarding schools promoted and managed the health and wellbeing of Indigenous secondary school students was by weaving relational networks to support student-centred healthcare and wellbeing, building rapport with Indigenous students as the basis for providing healthcare and wellbeing support, Developing relationships with families, discusses strategies of health care delivery more practically, conditions: professional and cultural capabilities of school staff, attracting and retaining professionally and culturally capable staff. Barriers to school staff capacity were high workloads, short-term contracts, and inadequate cultural proficiency. Being Indigenous to be an important quality needed in student support teams, Attracting and retaining professionally and culturally capable staff. Barriers to school staff capacity were high workloads, short-term contracts, and inadequate cultural proficiency.
Mcdonald [112]	2013	Examine how placements in community-based organisations enable trainee elementary school teachers to practice relationally	Trainee teachers	12	Interviews, focus groups, observations, and document review (also some surveys)	Comparative case analysis	Opportunities to 1) Develop deeper understandings of students and communities; (2) Develop more nuanced understandings of diversity, including intra-group diversity; (3) Examine school from an out-of-school perspective; and (4) Attend to the role of context in learning.
Meer [115]	2017	Describes the intricacy of familial relationships for women with intellectual disabilities in South Africa who experience gender-based violence	Service providers	58	Semi-structured interviews	Not mentioned	A social relational model of disability may be better able to include the role of families in the lives of women with intellectual disabilities
Miller [116]	2020	Explore practitioners’ views about the role of the narrative record in holding memories, feeding into recognition of capable agency, clarifying possibilities for action, restoration of identity and wellbeing	Carers	9 (six focus groups participants and 3 interviews)	Focus groups and in-depth interviews	Narrative hermeneutics	Though recording has come to be viewed as an onerous task by many practitioners, what emerged here was a stronger concern amongst practitioners to strike a balance between respectful interactions and flow of conversation and on the other hand, taking necessary steps within and between encounters to ensure that the record accurately reflects the carer perspective.
Moore [118]	2020	Explore what NHS mental health professionals value about the peer support worker role	Mental health professionals	5	Qualitative interviews	Discourse analysis, psychosocial theory	Mental health professionals valued peers for the deeply empathetic, relational approach they brought based on subjective experiences. Peer work was valued for the affect-focused quality of this work, and the challenge peers pose to existing values in mental health services. The values of peer support troubled dominant ways of working based in forms of knowledge that favour objectivity and hence encountered challenges.
Munford [122]	2020	Examine the experience of shame and recognition of vulnerable young people during transition to adulthood	Young people facing high levels of childhood adversity	107 in qual interviews (104 in final interviews)	Series of three interviews	Thematic analysis	Experiences of shame, misrecognition, and seeking recognition emerged as dominant themes in the young people's accounts. Young people's narratives also provided important insights into what constituted positive recognition. These were explored under each of the dimensions of recognition: love and care, respect, and being valued. The dimensions intersect and are realized within young people's trust‐based relationships with positive adults and peers.
Muusse [124]	2021	Describe dilemmas related to multiple perspectives on good community mental health care, using multiple stories about Building U.	Partners of the CMHT were selected based on the fieldwork. Additional interviews were also conducted with five team members to further clarify the first author's observations and to reflect on their work. At the end of the fieldwork, six service users known to the researcher from previous house visits were approached through their case manager for an interview about their experiences with care and support from the CMHT.	19	Participant- observation and standalone interviews	Qualitative analysis not specifically mentioned	In ordering care from a relational approach, the focus is not only on the individual, be it a citizen or a patient, but also on the relations between patients, caregivers, and others. Caring is working on these relations – trying to establish or maintain them – and with these relations – to avoid a crisis, there is no clear directive or general method that prescribes how these relationships should be crafted; it depends on circumstances, personal styles and who is involved. What is the good thing to do can differ along the way. In this mode of ordering care, it is impossible for care workers to only address mental illness as a discrete medical domain; they have to engage in non- medical domains of peoples’ life (work, finances, health). In doing this, the division between treatment and support becomes blurred. Good care is building trust, knowing people, and intervening when necessary, from within the established relationship.
Muusse [125]	2020	Exploring good care in the context of Trieste’s deinstitutionalised mental health care system/services	Not reported	Not reported	Interviews and participant observation	Qualitative exploratory	Good care involves working with and on relationships, care collective, negotiating goods, role of the professional "It’s about creating a social surrounding that functions as a buffer…. Working on the social determinants that create stability. Otherwise, the circle maintains itself.
Noseworthy [131]	2013	Critically explores current issues around decision-making and proposes a relational decision-making model for midwifery care.	Woman–midwife pairs in a large region in New Zealand in late 2009 and 2010. Women were 18 years or older, this was their first or subsequent pregnancy and they were between 34 and 37 weeks of gestation.	8	Interviews (prenatally and postnatally)	Thematic analysis	Themes included ontological and philosophical influences on decision-making; uncertainty, vulnerability, and relational trust; and socio-political and cultural influences. Inconsistencies in knowledge arising from social, cultural, and familial considerations as well as identities, beliefs, values, conversations, and practices were found to produce uncertainties around potential courses of action, expected consequences and outcomes. ‘Unplanned’ birth experiences decreased client autonomy and increased vulnerability thereby intensifying relational trust within decision-making. The political context may also open up or close down possibilities for decision-making at both national and local levels.
O'Meara [132]	2021	To explore women’s experiences of criminal justice systems to inform the development of guidance on working with women.	Study 1–women subject to community sentences or statutory licence periods ranging from 12 months to life sentences, all White British, mean age 41 years (SD = 10.34), the number of criminal convictions ranged from 1 to 22. Study 2–women currently serving sentences for violent or sexual offences, six identified their ethnicity as White-British, one as Asian-British, mean age was 35 years (SD = 5.1), number of criminal convictions held ranged from 1 to 53.	Study 1 = 6; study 2 = 7	Interviews	Interpretative phenomenological analysis and thematic analysis	Seven emergent themes indicated relational approaches to offender management may improve experiences of judicial systems for female offenders and for their probation officers. This approach may help prevent common systemic issues from perpetuating negative interactions between these groups. Specific suggestions for developing relational security and consistency of care within these relationships are provided.
Steckley [143]	2020	To identify and explore potential threshold concepts in residential childcare, with a corollary question about the utility of threshold concept theory in considering student and practitioner learning.	Educators and practitioners	Educators (*n* = 15) and practitioners (*n* = 14) and 7 practitioners recruited for follow-up	Focus groups and follow-up in-depth interviews	Qualitive data analysis	Relational practice was the most prominent potential threshold in terms of frequency, depth, and emphasis in discussions across the practitioner focus groups; it was discussed by all the groups.
Swan [145]	2018	Explores the psychodynamics of relationship-based practice from the perspective of young people in residential care.	Care leavers aged 18–24 years who had been in residential care in Ireland	10	Semi-structured interviews	Thematic analysis	Three themes emerged: first, the importance of a home-like environment; second, the positive elements of the key working relationship; and, finally, the importance of key working relationships enduring into the aftercare period. For many participants, their residential unit was the closest experience they had to a traditional concept of a home.
Townsend [148]	2020	Presents findings on the potential role of money as a mechanism to enhance these capabilities from an on-going evaluation of a major place-based initiative being implemented in 150 neighbourhoods across England: The Big Local (BL)	Diverse stakeholders, including residents and participant observation in a diverse sample of 10 BL areas as fieldwork sites	116 (interviews) participant observation (10 areas)	Interviews and participant observation	Thematic constant comparative approach	Money enabled the development of capabilities for collective control in these communities primarily by enhancing connectivity amongst residents and with external stakeholders. However, residents had to engage in significant ‘relational work ‘to achieve these benefits and tensions around the money could hinder communities’ ‘power to act’.
Tudor [150]	2020	Outlines some findings from an inquiry undertaken in the aftermath of 2011 earthquake in Christchurch, New Zealand, in which positive critique was used to examine the practice accounts of twelve school social workers alongside characteristics of recovery policies.	School social workers who had been working for a minimum of six months in schools in social work roles following the earthquakes, eight women and four men	12	Interviews	Foucauldian discourse analysis (FDA)	A feature of the participants’ accounts of their practices with affected school children in the recovery space is their commitment to restoring and protecting their clients’ well-being through therapeutically inclined relational practice. The notion of relationship primarily centred on the school social worker–client relationship, such that it was positioned as the means through which positive change occurred. The participants discussed their relational practices as a positive mode of practice to the extent that they positioned it as a form of empowerment for children in schools.
Valaitis [153]	2018	Examine Canadian key informants’ perceptions of intrapersonal (within an individual) and interpersonal (among individuals) factors that influence successful primary care and public health collaboration	Employed in or responsible for PC (*n* = 32; 43.2%), PH (*n* = 31; 41.9%), both sectors (*n* = 8; 10.8%) or neither sector (e.g., researchers) (*n* = 3; 4.1%), 5–40 years of healthcare experience, with 68% having over 20 years, most female (*n* = 58; 78.4%).	74	Interviews	Interpretive thematic analysis	Five interpersonal factors were found that influenced public health and primary care collaborations including: (1) trusting and inclusive relationships;(2) shared values, beliefs, and attitudes; (3) role clarity; (4) effective communication; and (5) decision processes. There were two influencing factors found at the intrapersonal level:(1) personal qualities, skills, and knowledge; and (2) personal values, beliefs, and attitudes.
Vielle [156]	2012	Examines the philosophy of justice embodied in tikanga Mãori, the Mãori traditional mechanism and approach to doing justice which adopts a holistic and relational lens, requiring that justice be seen in the context of relationships and crimes dealt with in terms of the relationships they have affected.	Nine participants were under the age of 30, and four were older than 60	33 Mãori	Interviews	Not mentioned	The Mãori approach to justice adopts a holistic and relational lens, which requires that justice be seen in the context of relationships and crimes dealt with in terms of the relationships they have affected. As a result, justice must be carried out within the community and the process owned by community members.
Ward-Griffin [157]	2012	Examines the provision of home-based palliative care for Canadian seniors with advanced cancer from the perspective of nurses.	Palliative care nurses	3	In-depth, semi-structured interviews and participant observations	Thematic analysis	Home-based palliative care nursing was depicted in this study as a dialectical experience, revealing three relational practice patterns: making time-forfeiting time, connecting-withdrawing, and enabling-disabling. Nurses attempted to negotiate the tensions between these opposing approaches to palliative care.
Wyness [167]	2016	School-participants’ perceptions and understandings of the social and emotional dimensions of schooling.	School staff and pupils aged between 11 and 17, parents	18 members of staff, 20 pupils, four parents.	In-depth interviews and focus groups	Explanatory interpretive case study	The emphasis on the relational and emotional work undertaken by teaching staff underpins the case-study school’s approach to challenging the barriers to learning. A number of themes and concerns are reported in this article including relational work in school that extends into the community, the school as a sprawling network of communication and the heighted role of the emotions at a number of levels in school. In drawing on interview data from teachers, school managers, pupils, and parents we are developing a model of schooling that approximates to Fielding’s conception of a people-oriented learning community.
*Quantitative*							
Andrews [13]	2018	Explore mothers’ service use at breaking the cycle, an early intervention and prevention program for pregnant and parenting women and their young children in Toronto, Canada.	Mothers with poly-substance use involved with child protection services (enrolled in service)	160	Secondary data analysis (client charts/notes and referral forms)	Statistical analysis (ANOVA, t-test)	These vulnerable women were actively engaged in many services and for a long duration, early engagement was associated with greater service use, and greater service use was associated with more positive circumstances upon ending service
Barrow [20]	2021	Service evaluation: explored viewpoints of key stakeholders, such as young people and frontline staff, about CSE services.	Young people experienced CSE, professional running CSE services	Young people *n* = 9; Professionals *n* = 9	Q-methodology	Q-sorts were subjected to factor analysis using Q-methodology software	Three factors were identified: (1) The importance of safety and atonement, (2) Managing trauma and mental health difficulties and (3) Family, normality, and a relaxed approach. All factors emphasized the importance of safety and trust between young people and professionals.
Emmamally [68]	2018	Describe the adherence of emergency healthcare professionals to family-centred practices in some emergency departments	Emergency department professionals	79	Survey using checklist of adherence to relational and participatory family-centred practices.	Statistical analyses included (ANOVA, t-test)	Family-centred practices are not consistently adhered to and do not feature in every family interaction. The majority of the participants treated families in a dignified and respectful manner but only 68% of the participants communicated clearly and so provided complete information at a level that families could understand.
Kuperminc [97]	2019	Examined associations among programmatic structures, workplace and workforce characteristics, and relational practices of program staff as they relate to young people’s ratings of their experience attending local clubs.	Ages 8–20 years old	57,710 members (aged 8–20) and 5231 staff	Member surveys	Independent samples t-tests, structural equation modelling	Strong correlations (.48 to .86) among the five indicators of relational practices as assessed at the setting level—establishing caring relationships, positive behaviour management, cultural sensitivity, setting high expectations, and youth input and agency—suggest a holistic view of staff interactions with youth: A staff that shows strength in one domain of relational practices is likely to have strengths in other domains.
Kutnick [98]	2014	Effectiveness of a relation-based group work approach adapted/co-developed by HK primary school mathematics teachers	Mathematics teachers (10 women and 10 men), pupils	20 teachers and 504 pupils	Survey and interactions observations	Descriptive analysis, parametric and non-parametric analysis	Use of the relational approach was also associated with an increase in the experimental teachers’ PEF scores, which is indicative of their greater enjoyment of teaching age-appropriate mathematics topics and of greater learning engagement by their pupils relative to the controls.
Laschinger [100]	2014	Test a model linking a positive leadership approach and work-place empowerment to workplace incivility, burnout, and subsequently job satisfaction	Nurses, 93.6% female, averaged 41.52 years of age, 16.80 years of nursing experience	1241	Survey	Structural equation modelling (SEM)	Resonant leadership had a strong positive direct effect on workplace empowerment (*β* = 0.47), which in turn had a significant negative effect on co-worker incivility (*β* = − 0.25). Resonant leadership also had a significant direct effect on job satisfaction (*β* = 0.16) and all indirect effects in the model were significant at the two-tailed *p* < 0.05 level.

Approaches used in the included mixed method studies varied widely (see Table 4) and often highlighted or focussed on the benefits of relational practice, rather than assessing effectiveness. Across the articles, the multi-component and complexity of relational practice is demonstrated (see Table 4).

Qualitative studies were mostly from health and social care/work sectors and highlight the time investment needed with relational approaches; noting the importance of staff motivation in order for relational practice to occur (see Table 4). The importance of trust was regularly mentioned across the articles and the importance of sharing stories and developing narratives, particularly around marginalisation to promote inclusion was highlighted.

Quantitative studies were fewer and varied; some focussed on examining the frequency or prevalence of relational practice and others examined the associated impacts (see Table 4).

A further 4 (2.53%) of the included publications were systematic or scoping reviews. There were two systematic reviews and one scoping review where relational practice was discussed. One systematic review synthesised qualitative research into older people’s experiences of acute healthcare [33], another synthesised research in adult acute inpatient mental health units, which focused on nurse-patient interaction [43] and a further systematic review used a meta-ethnography approach and explored studies of parent-school relations which impact positively on parents, regarding empowerment, parent voice and social capital [44]. The scoping review mapped health and social care and broader management literature to identify and extract important behaviours, processes and practices underlying the support of high-quality relationships [82].

### Definitions and underlying theories

The commonly used terms for defining relational practice and the underlying theories mentioned across the included articles are featured in Table 5.

**Table 5:** Relational practice terms used, with underlying theories, across the 4 sectors

Sector	Terms used for relational practice	Underlying theories mentioned
Criminal justice	Psychological-informed planned environments, therapeutic communities, pro-social environment, enabling environments, relational theory, relational model, relational justice	Psychoanalysis [18], trauma-informed [18], attachment [18], social psychological group theory [22], decolonising theories [25], positive psychology [28], Bourdieu’s concept of habitus [52, 177], relational authority [66], procedural justice [66], feminism [106], reintegrate shaming [156]
Education	Relational learning, relational approach, dialogic communication, relational framework, relational practice, relational framework, restorative practice, relational teacher development, relational pedagogy, relational theory, relationally focussed approach, teacher-student relationships, responsive classroom approach, relationship-resourced resilience model	Relational cultural theory [29], deliberate relationship model [29], relational communication theory [40], dialogue communication theory [40], ecological perspective [54], humanistic nursing [61], inclusive practices [65], constructivist self-determination theory [80], gestalt theory [90], relational field theory [90], humanistic psychology [94], Carl Rogers significant learnings [94, 175], psychoanalysis [123], trauma-informed approach [139], Gergen’s relational account of education [160, 164]
Health	Relational care, relational theory, relational approach, open dialogue, relational practice, relational stance, transformative communication, authentic partnership, family-centred care, relationally focussed leadership, relational focus, developmental-relational approach, relational perspective, enabling environments, relational ontologies, relational inquiry	Swanson’s Middle Range Theory of Caring [12, 178], conversational pedagogy [16], humanistic education [16], Carl Rogers Conditions theory [19, 175], systemic-relational perspective [19], compassionate healthcare [31, 32], normalisation process theory [32], Foucault meta-ethical framework [36], feminism [64], trauma-informed care [72], psychoanalysis [2], object relations [2], Boyatzis and McKee’s resonant leadership theory [100, 179], Kanter’s theory of organizational empowerment [100, 180, 181], Andersson and Pearson’s workplace incivility theory [100, 182], and Maslach and Leiter’s burn out theory [100, 183], motivational interviewing [120], socio-ecological model [133], ecological model [144], inter-organizational collaboration models developed by D’Amour et al. [153, 184] and San Martín-Rodríguez et al. [153, 185], Bourdieu’s theory of relational practice [153, 186], needs theorists (e.g., Henderson, Orem [165], Interaction theorist (e.g., Paterson and Zderad, Peplau [165], outcome theorists (e.g., Johnson, Rogers, Roy [165]. caring/becoming theorists (e.g., Watson and Parse [165]
Social work/care	Relational theory, relationship-based practice, guanxi, relational practice, family-centred inclusive practice, relational social work practice, relational autonomy, relational and strength approaches, restorative practice	object relations [17], attachment [17], self-psychology and relational psychoanalysis [17], critical theory (social work) [17], therapeutic and relationship-based model of care [34], social exchange theory [42], humanism [48], relational, systemic and complexity theories [71], Bourdieu relational theory [89, 186], trauma-informed care [170], feminism [138], relational theory of human connectedness [141], threshold concept theory [143], capabilities communities [148], emancipatory power framework [148], psychosocial perspective [149], psychodynamic perspective [150], capacity building approach [149]

The table shows that within the cross-sectoral terms being used to define relational practice that there are some certain commonalities, but equally there are different terms used aligned more to those specific sector types. For example, psychologically informed planned environments seem to be unique to criminal justice, relational learning and relational pedagogies to education, relational care to health and relational social work practices within social care. In contrast, enabling environments cross over criminal justice and health sectors, but are not mentioned within education or social work. Interpretation of the comparison of the cross-sector underlying theories however has fewer commonalities which is likely owing to the different sectors having more generally underpinning theoretical frameworks that are sector-specific.

### Reported impacts and benefits of relational practice

A total of 76 (48.10%) articles reported impacts and/or benefits of relational practice. Over half (*n* = 47, 61.84) of these articles noted workforce impact or client impacts (*n* = 41, 53.95%). A further 12 (15.79%) reported organisational/systemic impacts.

Health impacts could be seen across all sectors but rarely were they directly noted as a health impact, these have been broken down as workforce and client impacts.

In relation to workforce impacts, it is apparent that the workforce, when this way of working is embraced, benefit from the enhanced knowledge, insights, healthy working environments, and enhanced understanding of interpersonal dynamics. The use of relational practice also appears to enhance team cohesion and shared experiences [49, 63, 65, 72, 81, 89, 100, 118]. Some articles also reported skills enhancements from the adoption of relational practice with the enhancement of interpersonal skills including communication and empathetic listening [2, 34, 48, 50, 59, 60, 65, 72, 83, 85, 90, 101, 120, 139]. Personal benefits relating to confidence, increased employee satisfaction and more effective impact upon the progression and achievements of clients were also described [11, 12, 15–17, 20, 21, 26, 32, 33, 35, 58, 59, 63–65, 67, 72, 75, 76, 80–85, 90, 100, 108, 120, 125, 129, 130, 140, 145, 148].

Client health impacts included enhancement of wellbeing [16, 82, 139], physically, psychologically, socially, improved child custody [13] and physical health outcomes [74], mental health outcomes [81, 114, 139] and various educational attainments/outcomes [11, 18, 20, 24, 25, 75, 79, 80, 108, 140]. Recovery from client difficulties substance use difficulties, physical health, mental health and criminal justice outcomes were reported alongside enhanced interpersonal relationships with service providers and carers/families [44, 45, 63, 64]. Positive impacts of relational practice were reported in the reduction of health inequalities and engagement with society which included a reported sense of community belonging [28, 37, 86, 120]. From a health impact perspective, reports of reductions in trauma and re-traumatisation [81], emotional regulation skills and buffering of stress impacts were reported as was the reduction in re-offending and violent incidents in criminal justice contexts [21]. Promoting a sense of belonging was considered to have an important impact on health, and where relational approaches were not being used this could be overlooked [28, 139].

Whilst impacts from a more organisational perspective were less likely to be reported, when they were these referred to poignant and important learning and included the development of healthy sustainable communities [47, 129, 148]. The literature also referred to the development of insights into what works and what is important, with ideas shared such as the replacement of coercive controlling environments to negotiated engaged environments [50]. The invisibility of relational and interpersonal work was often described but its cruciality to working with people facing services and the importance of collaboration was also emphasised [48, 49, 85]. The relational practice was deemed to go beyond traditional working, having the potential to create environments with a focus on interagency working, emotional availability, enhancing the wellbeing of clients and the workforce alike.

## Discussion

This scoping of the literature identified a lack of articulation of a clear definition of relational practice within and across sectors. Rather, there is a more diffuse acknowledgement that various service provision settings enhance experiences and outcomes for the people who occupy these settings, be they designated as staff or service users. Whilst there is a view that there is an implicit knowledge of what relational practice is, this review demonstrates the lack of an explicit consensus and clarity across the sectors.

Whilst highlighting some commonalities in relation to what is meant by relational practice across the four sectors, the review also identifies certain unique differences. From an organisational level, there were very few explicit definitions of what relational practice is, or it consists of, but instead, a complex and multi-facetted complicated picture emerged that warrants some further synthesis using a different methodology, such as a conceptual review (currently being undertaken by the authors). There is a need for consensus and a unified model of understanding that Haigh and Benefield [3] have started to attend to, however, what has not been achieved previously is the scoping of the literature across the world and in the ambitious cross-sector approach we have adopted. This cross-sector approach adopted identified areas of agreement and conflict, but also enabled a shared learning approach to this important but lacking clarity concept of what relational practice is in an organisational context.

Equally through this review, we have discovered that whilst there are some sector-specific models of practice being proposed, there is a lack of empirical evidence relating to the implementation of relational practice and its effectiveness as we found no controlled trials and appropriately designed evaluation studies. Currently, most of the literature across the sectors identified was focussed upon opinion-based or qualitative studies. Owing to the lack of effectiveness trials and appropriately designed evaluations, we are also unable to examine impacts and effectiveness.

This review highlights that whilst the adopted use of ‘relational practice’ is increasingly being used in a variety of guises across sectors, the lack of definitional precision and clarity of understanding is problematic. There is however a sense that positive human relations can be aggregated within a setting harnessing a systematic, collective, rather than individual, approach to providing care, support or education across a variety of settings. Similarly, various theoretical perspectives are drawn upon without necessarily furnishing a unified conceptual basis for the described relational practice.

There is a tradition of practice-oriented rhetoric valuing so-called therapeutic relationships within health and social care systems, often associated with professional nursing [187] or social work practice [188]. More often than not, however, such commentary and theory-building are focused upon individual encounters rather than systemic interventions. A notable exception is the therapeutic community tradition that in turn developed out of a radical, innovative social psychiatric movement that has waned in influence in recent times but, nevertheless has offered an important legacy in initiatives such as psychologically informed planned environments (PIPES) [189] and the enabling environments kitemark in inpatient mental health services [190]. Recent scandals of abuse and neglect within inpatient mental health settings arguably expose the stark need for more organisationally defined approaches to therapeutic and supportive caring relations [191].

Albeit not exclusively, adult nursing appears more focused on technical aspects of care in contrast to forms of interpersonal and relational care more likely to be emphasised in mental health and addictions practice. The appeal of relational practice speaks to recent professional contestation of a perceived shift to the genericisation of mental health nurse education, where arguably core relational skills are poorly served in curricula that now prioritise adult nursing competencies [192]. In this regard, affinities for more relational approaches to care are associated with a preferred professional identity [193], and such affinities are often shared by service users seeking alternative forms of care and support to more narrowly defined bio-psychiatry, especially where this collides with coercive social control rather than consensual care and support [194].

### Limitations

Our findings represent the first scoping of the extant literature focusing on organisation and systemic relational practice across the four key sectors of service provision: health, social care, education and criminal justice. However, this review is not without limitations and hence the results whilst not conclusive should be also treated with caution. This is the first review to broadly scope relational practice across 4 sectors. There were clear overlaps across the sectors explored and a lack of clarity and consensus made this review challenging. The lack of a clear definition resulted in the broad search strategy and reaching a consensus within the research team at each stage required much deliberation.

We also chose to focus our review specifically organisational practice using a relational approach. We could equally have chosen to conduct this review from an individualised interaction perspective; however, to ensure that the review had focus and was feasible to conduct within our existing resources, we determined organisational and system-focussed relational practice would provide the greatest examples of this increasingly described way of working and enhancing practice in people-facing services.

Even with this narrowed focus, the size of the review across the 4 sectors remained ambitious and included a wide range of different types of publications. We were not able due to the scale of included papers to conduct a thorough quality appraisal as suggested by Levac et al. [195] enhanced scoping study methodology, so we have not reported on the quality/reliability of the articles included. There is also a risk of bias due to paper heterogeneity both within and across the disciplines; however, this can also be seen as a strength of the study as there are many benefits of adopting such a broad approach.

## Conclusions

The review shows that the concept of relational practice has good applied value, as evidenced by impact, yet the underpinning empirical evidence is limited and hence there is a need for clarity of understanding and more scientific effectiveness studies within this increasingly growing and important field of practice across sectors.

Ultimately, there is a need for conceptual standardisation of relational practice that draws upon the cross-sector intelligence. This in turn will facilitate a movement towards conceptual standardisation across the sectors. Conceptual inconsistencies hamper attempts to empirically investigate the relational practice constructs as is seen in the lack of research to test effectiveness, not least because this leads to a multitude of operationalisations of one construct.

This scoping review was necessary not merely to capitalise on what appears to be a cross-sector growing practice movement, which is highly valued, but also to avoid more ideologically based ways of practising which, potentially, are dependent on individual characteristics and intuition. It is perhaps not surprising that ‘relational practice’; however, it is defined and is highlighted in many papers as having organisational benefits. This scoping review has mapped available research on relational practice across four key fields. Despite certain definitional ambiguities and conceptual complexities, a number of positive benefits are claimed for the various relational approaches deployed within respective organisations.

### Supplementary Information

**Additional file 1.** Preferred Reporting Items for Systematic Reviews and Meta-Analyses extension for Scoping Reviews (PRISMA-ScR) Checklist.

**Additional file 2.** Full search strategy.

## Data Availability

The datasets used and/or analysed during the current study are available from the corresponding author on reasonable request.
