# Supplementary material for: Relational practice in health, education, criminal justice, and social care: a scoping review
Source: Syst Rev. 2023 Oct 13;12:194. doi: 10.1186/s13643-023-02344-9 (PMC10571424; doi:10.1186/s13643-023-02344-9)
Supplement: Supplementary file 2 — Additional file 2. Full search strategy. [file 13643_2023_2344_MOESM2_ESM.docx]

Additional File 2: Full Search Strategy

Criminal Justice Database

("psychosocial environments" OR "enabling environments" OR "Relational based" OR "Relational focussed" OR "Relational work" OR "Relational social work" OR "Relational centred" OR "Relational centered" OR "Relational practice*" OR "Relational informed" OR "Relational theory" OR "Relational approach" OR "Relational perspective*" OR "Relational model" OR "Relational strategy" OR "Relational strategies" OR "Relational Environment*" OR "Relational Justice*" OR "Relational Education*" OR "Relational Health*") OR ("Relational therapy" OR "Relational thinking" OR "Relational inquiry" OR "Relationship focussed" OR "Relationship based practice" OR "Relationship informed" OR "Interpersonal system*" OR "Interpersonal environment*" OR "Interpersonal practice" OR "Interpersonal approach*" OR "Interpersonal perspective*" OR "Interpersonal strategy" OR "Interpersonal strategies") AND (limited to Published Date: 2000/01/01-2021/12/31; Language: English)

Ovid Medline

Relational focus?ed.mp. OR Relational based.mp. OR Relational work.mp. OR Relational social work.mp. OR Relational centred.mp. OR Relational centered.mp. OR Relational practice*.mp. OR Relational informed.mp. OR Relational theory.mp. OR Relational approach.mp. OR Relational perspective*.mp. OR Relational model.mp. OR Relational strategy.mp. OR Relational strategies.mp. OR Relational Environment*.mp. OR Relational Justice*.mp. OR Relational Education*.mp. OR (Relational Health*.mp. OR Relational therapy.mp. OR Relational thinking.mp. OR Relational inquiry.mp. OR Relationship focus?ed.mp. OR Relationship based practice.mp. OR Relationship informed.mp. OR Interpersonal system*.mp. OR Interpersonal environment*.mp. OR Interpersonal practice.mp. OR Interpersonal approach*.mp. OR Interpersonal perspective*.mp. OR Interpersonal strategy.mp. OR Interpersonal strategies.mp. OR enabling environments.mp. OR psychosocial environments.mp.) AND (limited to (humans and year="2000 - 2021" and English)

EPIC

(“relational focussed” OR “relational practice*” OR “relational work” OR “relational social work” OR “relational centred” OR “relational centered” OR “relational informed” OR “relational theory” OR relational approach*” OR “relational perspective*” OR relational model” OR “relational strategy” OR relational strategies” OR “relational based”) OR (“relational environment*” OR “relational Justice*” OR “relational education*” OR “relational health” OR “relational therapy” OR “relational thinking” OR “relational inquiry”) OR (“relationship focussed” OR “relationship based practice” OR “relationship informed”) OR (“interpersonal system*” OR “interpersonal environment*” OR “interpersonal practice*” OR “interpersonal approach*” OR “interpersonal perspective*” OR “interpersonal strategy” OR “interpersonal strategies” OR “psychosocial environments” OR “enabling environments”) AND (limited to – Published Date: 2000/01/01-2021/12/31; Language: English)

SocIndex

(“relational focussed” OR “relational practice*” OR “relational work” OR “relational social work” OR “relational centred” OR “relational centered” OR “relational informed” OR “relational theory” OR relational approach*” OR “relational perspective*” OR relational model” OR “relational strategy” OR relational strategies” OR “relational based”) OR (“relational environment*” OR “relational Justice*” OR “relational education*” OR “relational health” OR “relational therapy” OR “relational thinking” OR “relational inquiry”) OR (“relationship focussed” OR “relationship based practice” OR “relationship informed”) OR (“interpersonal system*” OR “interpersonal environment*” OR “interpersonal practice*” OR “interpersonal approach*” OR “interpersonal perspective*” OR “interpersonal strategy” OR “interpersonal strategies” OR “psychosocial environments” OR “enabling environments”) AND (limited to – Published Date: 2000/01/01-2021/12/31; Language: English)

Criminal Justice Abstract

(“relational focussed” OR “relational practice*” OR “relational work” OR “relational social work” OR “relational centred” OR “relational centered” OR “relational informed” OR “relational theory” OR relational approach*” OR “relational perspective*” OR relational model” OR “relational strategy” OR relational strategies” OR “relational based”) OR (“relational environment*” OR “relational Justice*” OR “relational education*” OR “relational health” OR “relational therapy” OR “relational thinking” OR “relational inquiry”) OR (“relationship focussed” OR “relationship based practice” OR “relationship informed”) OR (“interpersonal system*” OR “interpersonal environment*” OR “interpersonal practice*” OR “interpersonal approach*” OR “interpersonal perspective*” OR “interpersonal strategy” OR “interpersonal strategies” OR “psychosocial environments” OR “enabling environments”) AND (limited to – Published Date: 2000/01/01-2021/12/31; Language: English)

Education Abstracts

(“relational focussed” OR “relational practice*” OR “relational work” OR “relational social work” OR “relational centred” OR “relational centered” OR “relational informed” OR “relational theory” OR relational approach*” OR “relational perspective*” OR relational model” OR “relational strategy” OR relational strategies” OR “relational based”) OR (“relational environment*” OR “relational Justice*” OR “relational education*” OR “relational health” OR “relational therapy” OR “relational thinking” OR “relational inquiry”) OR (“relationship focussed” OR “relationship based practice” OR “relationship informed”) OR (“interpersonal system*” OR “interpersonal environment*” OR “interpersonal practice*” OR “interpersonal approach*” OR “interpersonal perspective*” OR “interpersonal strategy” OR “interpersonal strategies” OR “psychosocial environments” OR “enabling environments”) AND (limited to – Published Date: 2000/01/01-2021/12/31; Language: English)

PsycInfo

(“relational focussed” OR “relational practice*” OR “relational work” OR “relational social work” OR “relational centred” OR “relational centered” OR “relational informed” OR “relational theory” OR relational approach*” OR “relational perspective*” OR relational model” OR “relational strategy” OR relational strategies” OR “relational based”) OR (“relational environment*” OR “relational Justice*” OR “relational education*” OR “relational health” OR “relational therapy” OR “relational thinking” OR “relational inquiry”) OR (“relationship focussed” OR “relationship based practice” OR “relationship informed”) OR (“interpersonal system*” OR “interpersonal environment*” OR “interpersonal practice*” OR “interpersonal approach*” OR “interpersonal perspective*” OR “interpersonal strategy” OR “interpersonal strategies” OR “psychosocial environments” OR “enabling environments”) AND (limited to – Published Date: 2000/01/01-2021/12/31; Language: English; Population Group: Human)

CINAHL

(“relational focussed” OR “relational practice*” OR “relational work” OR “relational social work” OR “relational centred” OR “relational centered” OR “relational informed” OR “relational theory” OR relational approach*” OR “relational perspective*” OR relational model” OR “relational strategy” OR relational strategies” OR “relational based”) OR (“relational environment*” OR “relational Justice*” OR “relational education*” OR “relational health” OR “relational therapy” OR “relational thinking” OR “relational inquiry”) OR (“relationship focussed” OR “relationship based practice” OR “relationship informed”) OR (“interpersonal system*” OR “interpersonal environment*” OR “interpersonal practice*” OR “interpersonal approach*” OR “interpersonal perspective*” OR “interpersonal strategy” OR “interpersonal strategies” OR “psychosocial environments” OR “enabling environments”) AND (limited to – Published Date: 2000/01/01-2021/12/31; Language: English; Population Group: Human)
